# The Design and Synthesis of Fluorescent Coumarin Derivatives and Their Study for Cu^2+^ Sensing with an Application for Aqueous Soil Extracts

**DOI:** 10.3390/molecules24193569

**Published:** 2019-10-02

**Authors:** Bin Qian, Linda Váradi, Adrian Trinchi, Suzie M. Reichman, Lei Bao, Minbo Lan, Gang Wei, Ivan S. Cole

**Affiliations:** 1 School of Engineering, RMIT University, GPO Box 2476, Melbourne, Victoria 3001, Australia; bin.qian@student.rmit.edu.au (B.Q.); suzie.reichman@rmit.edu.au (S.M.R.); lei.bao@rmit.edu.au (L.B.); ivan.cole@rmit.edu.au (I.S.C.); 2 CSIRO Manufacturing, Bayview Avenue, Clayton, VIC 3169, Australia; Adrian.Trinchi@csiro.au; 3 Shanghai Key Laboratory of Functional Materials Chemistry, School of Chemistry and Molecular Engineering, East China University of Science and Technology, 130 Meilong Road, Shanghai 200237, China; minbolan@ecust.edu.cn; 4 CSIRO Mineral Resources, PO Box 218, Lindfield, NSW 2070, Australia; Gang.Wei@csiro.au

**Keywords:** coumarin, Cu^2+^ sensing, fluorescent quenching, soil sensing

## Abstract

A series of fluorescent coumarin derivatives **2a**–**e** were systematically designed, synthesized and studied for their Cu^2+^ sensing performance in aqueous media. The sensitivities and selectivities of the on-to-off fluorescent Cu^2+^ sensing signal were in direct correlation with the relative arrangements of the heteroatoms within the coordinating moieties of these coumarins. Probes **2b** and **2d** exhibited Cu^2+^ concentration dependent and selective fluorescence quenching, with linear ranges of 0–80 μM and 0–10 μM, and limits of detection of 0.14 μM and 0.38 μM, respectively. Structural changes of **2b** upon Cu^2+^ coordination were followed by fluorescence titration, attenuated total reflection Fourier transform infrared spectroscopy (ATR-FTIR), mass spectrometry, and single crystal X-ray diffraction on the isolated Cu^2+^-coumarin complex. The results revealed a 1:1 stoichiometry between **2b** and Cu^2+^, and that the essential structural features for Cu^2+^-selective coordination are the coumarin C=O and a three-bond distance between the amide NH and heterocyclic N. Probe **2b** was also used to determine copper (II) levels in aqueous soil extracts, with recovery rates over 80% when compared to the standard soil analysis method: inductively coupled plasma-mass spectrometry (ICP-MS).

## 1. Introduction

Heavy metal contamination [1] in soil and water is a major concern due to its direct effect on the quality of drinking water, safe food production [2,3], and the detrimental impact on human health when present in the body in excessive amounts [4,5]. However, heavy metals are also important micronutrients for living organisms, thus are desired ingredients in soil and water [6]. Copper is known as one of the most abundant essential trace elements in human body [7,8], and is also required by many living organisms to maintain healthy physiological processes due to its redox-active nature [9]. The World Health Organization (WHO) sets the maximum allowable level of Cu^2+^ in drinking water at 30 μM and recommends that the population mean intake should not exceed 10–12 mg per day for adults [10]. Thus, the development of methods allowing for sensitive, selective, and real-time detection of bioavailable heavy metals, including Cu^2+^, in environmental samples is ever relevant.

Some of the most readily utilized sensing methods are based on molecular probes that display concentration dependent changes in their optical properties arising from the structural changes upon interaction with the analytes of interest [11]. Coumarins are attractive for use as fluorescent optical probes, due to their easy-to-tailor structure that allows for metal ion-selective coordination sites, as well as their exhibiting significant changes in their optical signals upon interaction with such target analytes [12]. When interacting with Cu^2+^, coumarins can undergo ligand to metal charge transfer, forming a Cu^2+^–coumarin complex. This can inhibit the intramolecular charge transfer (ICT) – the mechanism responsible for its fluorescence – within the coumarin. Thus, complex formation can result in the quenching of the coumarin’s fluorescence [13,14,15,16]. A broad range of coumarin-based fluorescent Cu^2+^-sensors have been reported [17]. For example, a ratiometric coumarin-hydrazone sensor exhibited Cu^2+^ selectivity in the presence of other transition metal ions, and its fluorescence emission at 574 nm was quenched by increasing amounts of Cu^2+^ present [18]. A reversible ratiometric Cu^2+^-sensor incorporating two covalently linked fluorophores (coumarin and 4-amino-7-sulfamoyl benzoxadiazole) displayed Cu^2+^-induced blue shift of its emission (from 555 to 460 nm) upon coordination of Cu^2+^ due to decreased intramolecular fluorescence resonance energy transfer (FRET) [19]. Despite the ever-increasing number of optical molecular probes reported for Cu^2+^ detection, improvements in selectivity and sensitivity, and a better understanding of the structural requirements when designing these Cu^2+^-coordinating molecular probes, remain of interest.

**Scheme 1:**
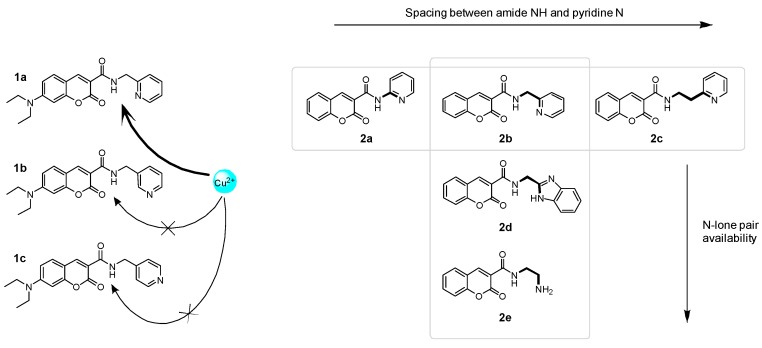
Schematic representation of (**a**) the Cu^2+^-coordinating capability of picolyl-substituted diethylamino-coumarins **1a**–**c**, and (**b**) the newly designed coumarins **2a**–**e**.

This work was aimed at the design, synthesis, and simultaneous evaluation of a series of structurally related coumarin derivatives to further understand the structural requirements towards optical Cu^2+^-sensors that have enhanced selectivity and sensitivity, especially when employed in mixed aqueous media. Previously reported work on picolyl-substituted coumarin-3-carboxylic acid derivatives **1a**–**c** (Scheme 1a) [20] indicated that the distance and relative orientation of the pyridine nitrogen relative to the other coordinating moieties (the coumarin C=O and the amide NH) were essential features affecting the ability to coordinate Cu^2+^. Coumarin **1a** displayed Cu^2+^-selective fluorescence quenching, as opposed to **1b**–**c** which failed to do so. Herein, we designed a series of coumarin derivatives **2a**–**e** (Scheme 1b) incorporating various spacers between the amide NH and a pyridine N (two-bond in **2a**, three-bond in **2b**, and four-bond in **2c**), or benzoimidazole N in **2d**, or ethylenediamine -NH_2_ in **2e**. Thus, information on the effect of (i) the different spacers between the Cu^2+^-coordinating heteroatoms in **2a**–**c**, and (ii) the distribution and availability of the electrons on the nitrogen oriented three-bonds away from the amide NH (**2b**, **2d**, and **2e**) on the coordination of Cu^2+^ can be gained. The coumarin derivatives which showed Cu^2+^-dependent fluorescence quenching were further studied by attenuated total reflection Fourier transform infrared spectroscopy (ATR-FTIR), mass spectrometry, and single crystal X-ray diffraction to indicate changes in their structure before and after Cu^2+^-coordination. From the results obtained, candidate **2b** was evaluated on aqueous extracts of real soil samples collected from the roadside in the western parts of Melbourne, Victoria, Australia; the results were compared to the standard, inductively coupled plasma mass spectrometry (ICP-MS) method.

## 2. Results and Discussion

### 2.1. Design and Synthesis of Coumarin Derivatives **2a**–**e**

In order to gain further information on the ideal relative atomic arrangement of the Cu^2+^-selective coordinating moiety within substituted carboxylic coumarins; a series of alkylamino-pyridines **3a**–**c**, a benzimidazole **3d**, and a linear chain mono-protected diaminoethylene **3e** were selected and subjected to amide bond formation with coumarin-3-carboxylic acid using PyBOP as a coupling agent and triethylamine as a non-nucleophilic base (Scheme 2) [21]. Although the yields of **2a**–**e** were moderate (Table 1) (presumably due to the potential side reactions resulting in undesired by-products) [22], the choice of this coupling agent allowed for a facile, one-step purification process upon completion of the reaction. Due to the formation of water-soluble by-products, the desired products **2a**–**d** and Boc**-2e** were isolated after a simple extraction to a purity suitable for characterisation and analytical studies. Boc-**2e** was then subjected to deprotection using TFA, and then the TFA salt of **2e** was neutralised with NaOH to obtain **2e**. The resulting coumarins **2a**–**e** were characterised by ^1^H and ^13^C-NMR, IR, and high-resolution mass spectrometry (Figures S1–S20). Absorption, and fluorescence excitation and emission spectra for **2a**–**e** were recorded and the corresponding maxima are listed in Table 1. The Stokes shifts (∆ = λ_em_ − λ_ex_) were greater than 100 nm for each of these coumarin derivatives **2a**–**e. This** is a desired property for optical molecular probes, facilitating enhanced sensitivity due to the lesser overlap between the selected wavelengths of the exciting light-source and the emission filter in the detector recording the sensing signal [23].

### 2.2. The Effects of pH and Concentration On The Fluorescence of **2a**–**e**

**Figure 1:**
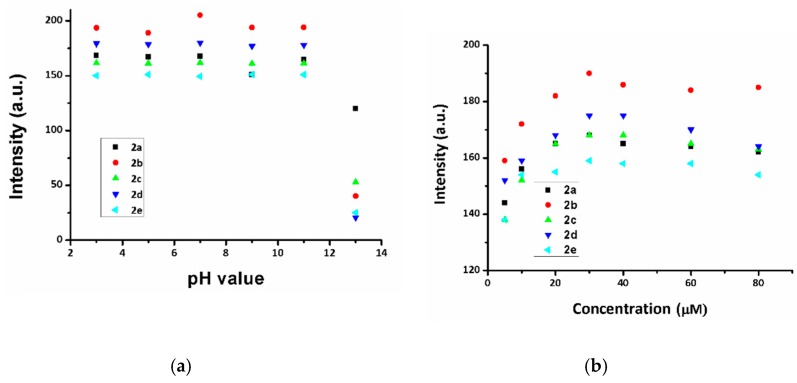
Fluorescence emission intensities of **2a**–**e** (λ_ex_ according to Table 1) (**a**) at various pH values and (**b**) at various dissolved coumarin concentrations in DMSO/HEPES buffer (*v*/*v*, 1/9).

In order to find the optimum conditions to evaluate the Cu^2+^-sensing performance, fluorescence intensities of **2a**–**e** were recorded across the pH range of 3–13 (Figure 1a). The fluorescence of **2a**–**e** between pH 3–11 showed no significant changes; however, above pH 11, sharp quenching was observed. Fluorescence intensities at various concentrations of dissolved coumarin **2a**–**e** were also recorded within the range of 5–80 µM (Figure 1b). For the subsequent studies, 30 µM of dissolved **2a**–**e** at pH 7 was chosen, as beyond that concentration, no further concentration dependent fluorescence was observed.

### 2.3. UV-Vis and Fluorescence Properties of **2a**–**e** in the Presence of Cu^2+^

**Figure 2:**
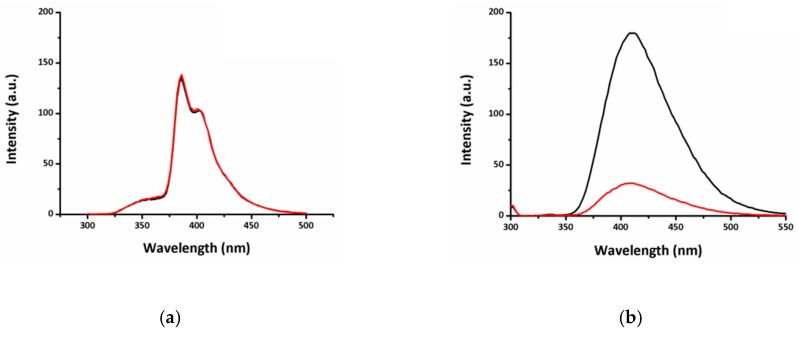

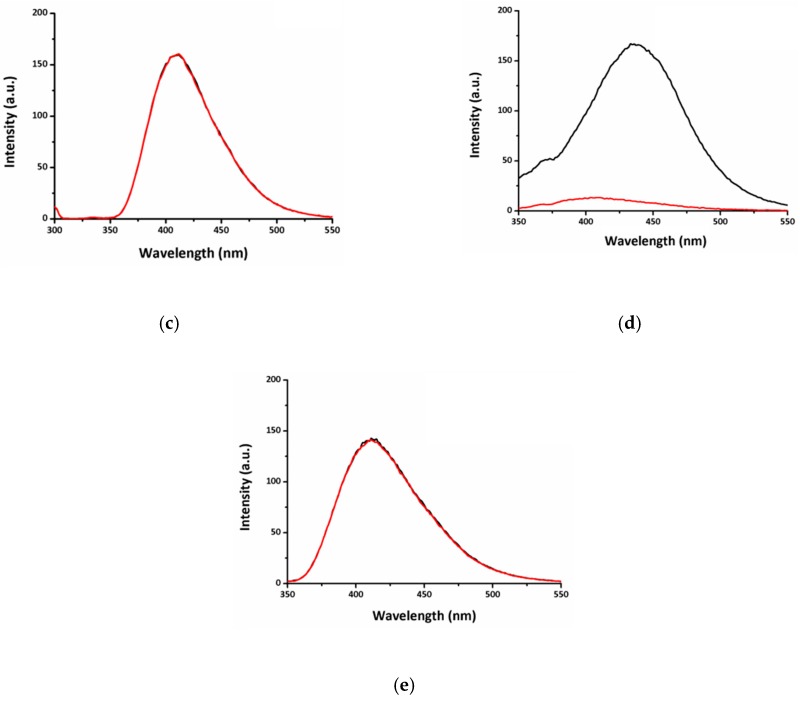
Fluorescence intensities of **2a**–**e** (30 µM) in DMSO/HEPES buffer (*v*/*v*, 1/9) in the absence (black lines) and presence (red lines) of one equivalent of CuCl_2_ at their corresponding excitation maxima (see Table 1) (**a**) **2a**; (**b**) **2b**; (**c**) **2c**; (**d**) **2d**; (**e**) **2e**.

UV-Vis spectra of **2a**–**e** in DMSO/HEPES buffer (*v*/*v*, 1/9) were recorded upon the addition of one equivalent of CuCl_2_ (Figure S21). Coumarins **2a** and **2c**–**e** showed no changes in their absorbances, with only **2b** showing a broadening of the absorption peak upon addition of CuCl_2_. Recording of the fluorescence emission intensities of **2a**–**e** in DMSO/HEPES buffer (*v*/*v*, 1/9) in the absence and presence of one equivalent of CuCl_2_ (λ_ex_ according to Table 1) revealed that **2a**, **2c**, and **2e** exhibited no optical response to Cu^2+^ (Figure 2). However, fluorescence quenching was observed for both **2b** (λ_em_ = 412 nm) and **2d** (λ_em_ = 436 nm), suggesting the coordination of Cu^2+^. In **2b**, **2d**, and **2e**, the relative arrangement of the moieties that can donate electrons towards the copper are similar, yet despite this, only **2b** and **2d** exhibited optical responses upon interaction with the Cu^2+^. The lack of fluorescence quenching in **2e** is proposedly due to the greater flexibility of the linear chain amino group that does not facilitate the formation of a metal-coumarin complex. Based on these results, a three-bond spacing and restricted flexibility between the heterocyclic nitrogen and the amide NH are deemed to be essential for the Cu^2+^-based fluorescence quenching, and consequently, **2b** and **2d** were further studied for their Cu^2+^-sensing performance is aqueous media.

**Figure 3:**
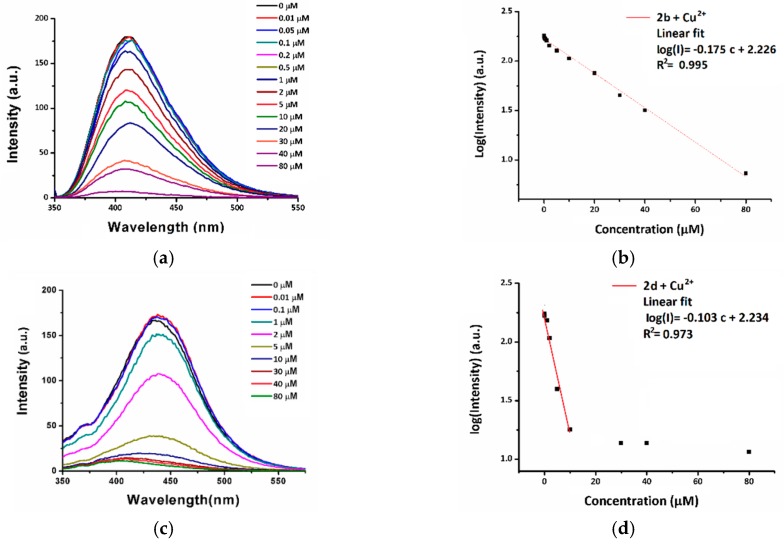
Fluorescence spectra of (**a**) **2b** (λ_ex_ = 303 nm) and (**c**) **2d** (λ_ex_ = 325 nm) (30 µM) with the addition of various concentrations of CuCl_2_ in HEPES/DMSO buffer and the corresponding titration curves and linear ranges of (**b**) **2b** and (**d**) **2d.**

Firstly, fluorescence titrations of **2b** and **2d** (30 µM) with CuCl_2_ (0–80 µM) in DMSO/HEPES buffer were carried out (Figure 3) to identify the linear range and the limit of detection (LOD). Both **2b** and **2d** responded with gradually quenched fluorescence intensities at their emission maxima, 412 nm and 436 nm, respectively, during the addition of increasing amounts of CuCl_2_. The fluorescence quenching of **2b** by Cu^2+^ was linear (R^2^ = 0.995) within the Cu^2+^ concentration range of 0–80 µM, which is a significant improvement from the previously reported probe **1a**, whose response was linear only within the range of 0–25 μM [20]. The calculated limit of detection (LOD) was 0.14 µM (LOD = 3σ/slope, where σ is standard deviation of the blank measurements) [24]. Both the linear range and the LOD were satisfactory to fulfil the sensing requirements for monitoring copper levels in drinking water, as the maximum allowed level is specified at 30 µM by the Australian Drinking Water Guidelines [25]. Meanwhile, **2d** (Figure 3c–d) exhibited a rather narrow linear range of 0–10 µM, and a calculated association constant of 7.83 × 10^5^ M^−1^ (versus that of **2b** at 6.85 × 10^4^ M^−1^) [26], and a LOD of 0.38 µM. Furthermore, a shift of the emission maxima from 436 nm towards 405 nm was observed in the presence of Cu^2+^ at concentrations >10 µM. Thus, **2d** may only be applicable for Cu^2+^-sensing within the range of 0.4–10 µM. Also, the greater association constant shows higher affinity of **2d** to coordinate Cu^2+^, which is suggested to be indicative to the greater availability of the heterocyclic N lone pair in **2d** [27], hence the faster coordination of Cu^2+^.

For both **2b** and **2d**, the fluorescence quenching mechanism can be explained by the previously reported ligand to metal charge transfer [28]. The fluorescence quenching results herein showed that a decrease of the emission intensity occurs beyond the addition of one equivalent (30 μM) of Cu^2+^ to both **2b** and **2d** (Figure 3a,c). This phenomenon was previously studied via femtosecond time-resolved fluorescence, showing evidence that the charge transfer only contributes to 29% of the overall fluorescence quenching [20].

**Figure 4:**
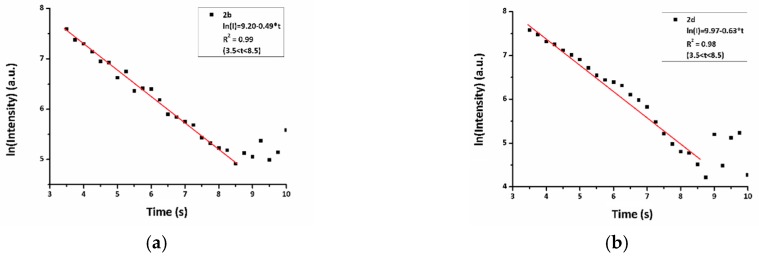
First-order plots for the coordination of Cu^2+^ with (**a**) **2b** and (**b**) **2d.**

To further assess the time-frame of developing coordination and charge transfer between Cu^2+^ and the probes, the changes in the fluorescence intensity as functions of time were recorded for **2b** and **2d**. After the addition of two equivalents of Cu^2+^ (ensuring the full quenching of fluorescence), and a subsequent mixing step taking 3.5 sec, the fluorescence intensity values were recorded at 25 ℃ (Figure 4) and fitted with modified first order kinetics according to (Equation (1)) [29] to identify the time points where the fluorescence was quenched by the Cu^2+^:

(1) ln(I)= k × t + b

where I (a.u.) was fluorescence intensity of the coumarins, t (s) was time, and k (s^−1^) was the rate constant of the first-order model. The linear range of ln(I) versus time (s) function for **2b** and **2d** is shown in Figure 4, with R^2^ values of 0.99 and 0.98, respectively. To determine the time frame within which the fluorescent intensities of **2b** and **2d** were quenched by the copper, firstly, the average quenched fluorescence intensity was identified, as shown in Figure S22. Then, based on the linear fitted equations (Figure 4), the quenching time points were calculated to be 7.5 sec and 6.9 sec for **2b** and **2d**, respectively. Those results show the faster quenching of **2d** compared to **2b**, suggesting greater availability of the heterocyclic N lone pair in **2d** [27] resulting in faster coordination.

**Figure 5:**
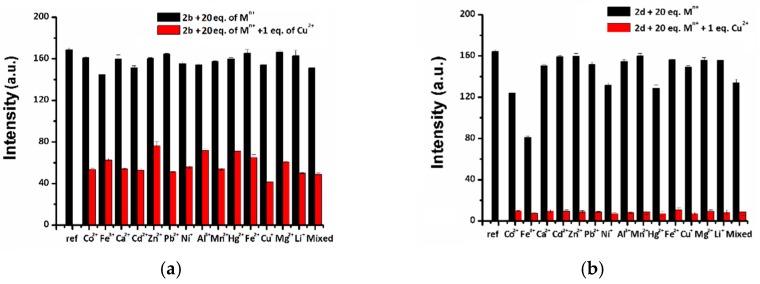
Fluorescence emission intensities of solutions containing 20 eq. of various cations and (**a**) **2b** and (**b**) **2d** (both 30 μM) in the presence (red bars) and absence (black bars) of 1 eq. of Cu^2+^ in DMSO/HEPES buffer.

One of the most indispensable qualities of a fluorescent probe is its selectivity towards a target analyte over other commonly encountered species. To evaluate the selectivity of **2b** and **2d** (30 μM) towards Cu^2+^ (30 μM), a variety of metal ions (30 μM) including Al^3+^, Ca^2+^, Cd^2+^, Co^2+^, Fe^3+^, Mn^2+^, Pb^2+^, Hg^2+^, Ni^2+^, Cu^+^, Mg^2+^, Li^+^, and Zn^2+^ were pre-mixed with the probes and fluorescence emissions were examined with and without the addition of Cu^2+^. In the presence of one equivalent of the potentially interfering cations, both **2b** and **2d** exhibited selectivity towards Cu^2+^ (Figure S23). When considering real life assays, the copper may be present at a significantly lower concentration compared to other metal ions; therefore, fluorescence quenching in the presence of excess amounts (20 equivalents) of interfering metal ions was also investigated for **2b** and **2d** (Figure 5). Fluorescence intensity prior the addition of the target analyte Cu^2+^ was quenched by more than 10% by only Fe^3+^ and Cd^2+^ for **2b** (Table S1), and Co^2+^, Fe^3+^, Ni^2+^, and Hg^2+^ for **2d** (Table S2). The inferior selectivity observed in the case of **2d** suggests that the heterocyclic N and the availability of its lone pair have an essential role in the coordination of the metal ion species.

**Figure 6:**
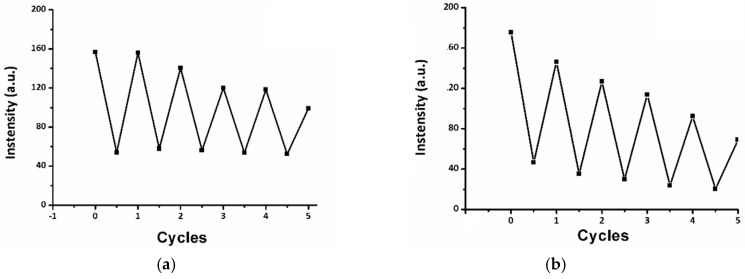
Fluorescence intensity of (**a**) **2b** and (**b**) **2d** upon the consecutive addition of Cu^2+^ followed by EDTA for five cycles in DMSO/HEPES buffer.

Another desirable property of a sensing system is the potential for repeated recovery of the sensor, which results in an extended life-cycle and applicability. Cu^2+^ is known for its high affinity towards chelating agents, such as L-cysteine [30], EDTA [31], and dopamine [32]. In this work, EDTA was used to record fluorescence recovery of both **2b** and **2d**. Upon the alternating addition of 1 eq. of Cu^2+^, then EDTA, the fluorescence levels of **2b** was recovered up to approximately 80% of its original intensity through four recovery cycles (Figure 6). These results indicated that **2b** could be employed in reusable optical sensing devices for Cu^2+^ detection.

A summary of the sensing properties for **2b**, **2d**, and the previously published probe **1a**, can be found in Table 2. It is apparent that **2b** shows improvements both in its LOD and linear range for Cu^2+^-sensing in DMSO/HEPES buffer.

### 2.4. The Coordination of Cu^2+^ by Coumarin **2b**

**Figure 7:**
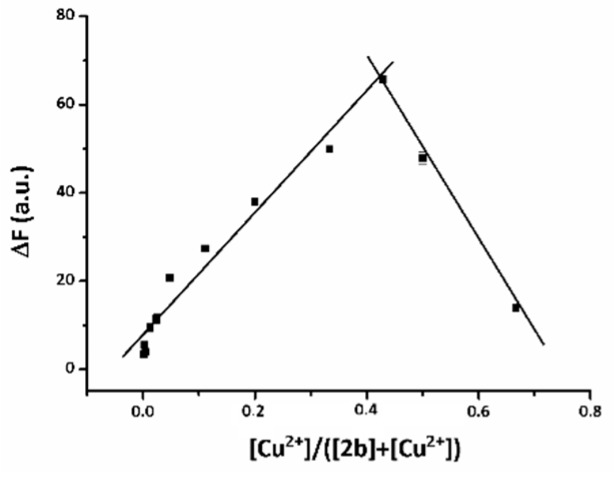
Job plot based on the fluorescence titration of **2b** (30 µM) with Cu^2+^.

The fluorescence titration of coumarins **2a**–**e** showed that **2b** and **2d** had affinities to coordinate Cu^2+^, with **2b** having a suitable linear range and selectivity for practical application. Hence, the interaction between **2b** and Cu^2+^ was further studied. Job plot, solid state ATR-FTIR, single crystal X-ray diffraction, and mass spectra were recorded on **2b** and the isolated **2b**–Cu^2+^ complex. The Job plot analysis, based on the fluorescence recorded by titrating **2b** with Cu^2+^ (Figure 7), revealed a 1:1 stoichiometry between **2b** and Cu^2+^. This corresponds with the mass spectra depicting a peak for **2b**-Cu^2+^ complex at *m/z* 383.03 (Figure S24). (Note that the corresponding peak is due to the association of the complex with the solvent used, acetonitrile.) However, tridentate chelation [33] of Cu^2+^ by **2b** in a 1:2 stoichiometry is apparent by a *m*/*z* 622.09 peak in the mass spectra (Figure S24); upon increasing Cu^2+^ concentration this form transitions into a 1:1 complex.

**Figure 8:**
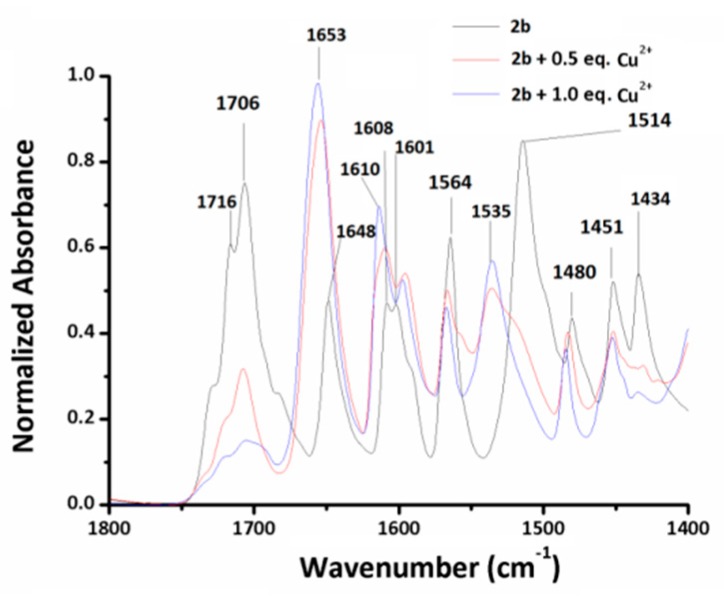
Normalized ATR-FTIR spectra of **2b** (black line) and **2b**-Cu^2+^ complex formed upon the addition of 0.5 eq. (red line) and 1 eq. (blue line) of CuCl_2_ to **2b** (30 μM) in DMSO/HEPES buffer.

Infrared spectra were also recorded on the solids isolated from an acetonitrile solution of **2b** in the absence and presence of added Cu^2+^ (Figure 8 and Figure S25). Notably, the solid samples isolated from acetonitrile were a mixture of **2b**, and **2b**–Cu^2+^. By overlapping the normalised IR spectra, the characteristic changes in the intensities of particular peaks have confirmed the previously reported observation, that the key Cu^2+^-coordinating moieties are the coumarin C=O, the amide NH, and the pyridine N (Table 3). 

Firstly, the defined peak at 3301 cm^−1^ corresponding to the NH stretch in **2b** became broader and less intense upon the coordination of Cu^2+^ (Figure S25). The most apparent changes occurred within the region of 1680–1750 cm^−1^ originating from alterations of the coumarin C=O bond within **2b**. Upon coordinating the Cu^2+^, the coumarin C=O peak at 1706 cm^−1^ became significantly less intense, suggesting the involvement of the oxygen lone pair electrons in the coordinative bond. Additionally, the amide C=O stretch shifted from 1648 in **2b** to 1653 cm^−1^ in **2b**-Cu^2+^ along with increased peak intensity. That suggests a lesser role of the carbonyl moiety in the potential amide resonance structures, hence the restoration of the amide C=O double bond, resulting in a stronger dipole, thus absorption at higher wavenumbers. Further changes were observed in the peaks presenting at about 1608 cm^−1^, depicted as C=N stretches of the pyridine ring. Both the amide NH and C–N peaks at 1564 cm^−1^ decreasing in intensity, and the transition of the 1514 cm^−1^ peak of free ligand **2b** into 1550 cm^−1^ was observed (see shoulder of **2b**-Cu^2+^) upon coordination with the Cu^2+^. Comprehensive loss of those peaks was not expected for two reasons: firstly, the studied solids were a mixture (as discussed above); secondly, the peak at 1564 cm^−1^ originates from both the vibration of amide NH and the stretch of amide C–N bonds. Furthermore, upon addition of Cu^2+^, the peak at 1451 cm^−1^ was reduced in intensity which suggested changes of the possible resonance structures in the pyridine ring.

**Figure 9:**
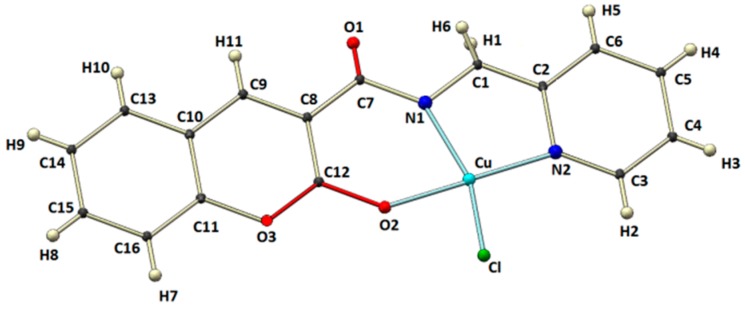
Single crystal structure of **2b**-Cu^2+^.

Ultimately, single crystal X-ray diffraction was recorded on the crystal grown from an aqueous acetonitrile solution containing **2b** and CuCl_2_ in a 1:1 molar ratio (Figure 9, Figure S26, Tables S1 and S2), as well as single crystal of **2b** itself. The as-observed structure of **2b**-Cu^2+^ complex is depicting a 1:1 stoichiometric ratio, and that the coumarin O(3), the amide N(2), the pyridine N(1), and a residual chlorine Cl(1) are the coordinating moieties [20]. This four-bond coordination was previously explained by the sp^3^ hybridization of Cu^2+^ when dissolved in acetonitrile water mixture [34]. According to the structure exhibited in the X-ray diffraction, the hydrogen of amide N(1) is displaced during the coordination process. Moreover, the bond length of coumarin C=O increased from 1.21 Å to 1.35 Å upon Cu^2+^ coordination, which falls between the conjugated and single bond length [35,36] (Table 4). This correlates with the IR results, translating into the diminishing C=O IR peak at 1707 cm^−1^ upon increasing Cu^2+^ concentration (Table 3). 

### 2.5. The Evaluation of **2b** on Soil Samples

The evaluation of **2b** for Cu^2+^-sensing in soil was conducted using a water extraction [37] of roadside soil samples collected in the western parts of Melbourne, Victoria, Australia. Fluorescence titrations generated using **2b** were compared against the industry standard of ICP-MS quantification [38]. ICP-MS quantifies elemental total copper content, while use of **2b** measures dissolved Cu^2+^; comparison is still feasible, as it is accepted within the field that following sample preparation, most copper species will be present as Cu^2+^ [37,39,40]. The standard curve of **2b** was recorded (Figure S27) and background fluorescence of soil extracts was also tested (Figure S28), allowing for correction when calculating the Cu^2+^ concentration by **2b**. Recovery of Cu^2^^+^, directly from the water extraction without further sample manipulation was >80% for all three soil samples tested (Table 5).

## 3. Materials and Methods 

### 3.1. Materials and Instruments

Benzotriazol-1-yloxy tripyrrolidinophosphonium hexafluoro-phosphate (PyBOP) was purchased from Oakwood Chemical (West Columbia, South Carolina, SC, USA). Coumarin-3-carboxylic acid (99%), 2-amino pyridine (99%), 2-(2ʹ-pyridyl)ethylamine (95%), and trifluoroacetic acid (99%) were purchased from Sigma-Aldrich (St. Louis, MO, USA). 2-(Aminomethyl)pyridine (99%) was obtained from Acros Organic (Belgium, WI, USA). 2-(Aminomethyl)benzimidazole dihydrochloride (98%) was purchased from Alfa Aesar (Ward Hill, USA). *N*-Boc-ethylenediamine (98%) was purchased from Combi-blocks (SanDiego, CA, USA). All chemicals were used as received without further purification. IR spectra were recorded on Nicolet 6700 FT-IR (Thermo Scientific, Waltham, MA, USA). UV-Vis and fluorescence spectra were measured on Varian Cary Eclipse 5G UV-Vis-NIR spectrometer and Varian Cary Eclipse fluorescence spectrophotometer (CMUSA), respectively. NMR were recorded on Bruker Ascend (Billerica, MA, USA) at 400 MHz for ^13^C and 100 MHz for ^1^H, respectively. Mass spectra were obtained on Thermo Scientific Q Exactive spectrometer in high resolution electrospray mode. Single crystal X-ray diffraction was recorded on Bruker Axs Apex Duo Single crystal XRD instrument. ICP-MS was conducted on Agilent Technologies 7700x ICP-MS Analyser (Santa Clara, CA, USA). CLARIOstar plate reader was used for kinetics study.

### 3.2. General Synthetic Method for the Preparation of **2a**–**e**

Coumarin derivatives **2a**–**e** were synthesised by the adaptation of a previously reported procedure with an additional step of deprotection for Boc-**2e** [20]. Coumarin-3-carboxylic acid (1 eq.), a selected amine (1.1 eq.), and PyBOP (1 eq.) were stirred at room temperature in acetonitrile. Upon completion, the reaction was quenched with brine (30 mL/5 mmol of coumarin-3-carboxylic acid) and extraction was conducted with dichloromethane (30 mL/5 mmol of coumarin-3-carboxylic acid). The organic layer was then washed with 10% citric acid, then 10% NaHCO_3_, following water and brine. The organic residues were dried over Na_2_SO_4_. Finally, the solvent was removed under vacuum. The residues were triturated with ethyl acetate to give the desired product as a solid.

#### 3.2.1. Synthesis of 2-oxo-*N*-(pyridin-2-yl)-2H-chromene-3-carboxamide (**2a**)

Yield of **2a**: 26%; white solid; FTIR (neat, cm^−1^) C=O 1701, N–H 3261 and 1565, C=N 1670, C–N 1534; ^1^H-NMR (400 MHz, DMSO-*d_6_*, ppm): *δ* 7.20 (1H, t, *J* = 6.1 Hz, CH-4ʹ), 7.49 (1H, t, *J* = 7.6 Hz, CH-7), 7.57 (1H, d, *J* = 8.4 Hz, CH-8), 7.81 (1H, t, *J* = 7.8 Hz, CH-6), 7.89 (1H, t, *J* = 7.8 Hz, CH-5ʹ), 8.07 (1H, d, *J* = 7.8 Hz, CH-5), 8.27 (1H, d, *J* = 8.3 Hz, CH-3ʹ), 8.39 (1H, d, *J* = 4.9 Hz, CH-6ʹ), 9.05 (1H, s, CH-4), 11.17 (1H, s, NH). ^13^C-NMR (100 MHz, CDCl_3_, ppm): *δ* 115.0, 116.9, 118.5, 118.7, 120.4, 125.5, 130.1, 134.7, 138.3, 148.5, 149.4, 151.3, 154.8, 159.9, 161.3; LRMS ASAP [**2a** + H^+^] 267.07; HRMS ASAP, [**2a** + H^+^] calculated *m*/*z* C_15_H_11_N_2_O_3_ 267.0725, found = 267.0764.

#### 3.2.2. Synthesis of 2-oxo-*N*-(pyridin-2-ylmethyl)-2H-chromene-3 carboxamide (**2b**)

Yield of **2b** 23%; white solid; FTIR (neat, cm^−1^) C=O 1706, N–H 3301 and 1564, C=N 1648, C–N 1514; ^1^H-NMR (400 MHz, DMSO-*d_6_*, ppm): *δ* 4.65 (2H, d, *J* = 5.6 Hz, CH_2_), 7.28 (1H, t, *J* = 6.2 Hz, CH-4ʹ), 7.39 (1H, d, *J* = 7.8 Hz, CH-3ʹ), 7.46 (1H, t, *J* = 7.5 Hz, CH-7), 7.53 (1H, d, *J* = 8.4 Hz, CH-8), 7.77 (2H, m, CH-5ʹ and 6), 8.01 (1H, d, *J* = 7.2 Hz, CH-5), 8.55 (1H, d, *J*=4.9 Hz, CH-6ʹ), 8.91 (1H, s, CH-4), 9.44 (1H, t, *J* = 5.5 Hz, NH); ^13^C-NMR (100 MHz, CDCl_3_, ppm): *δ* 45.5, 116.7, 118.5, 118.7, 121.8, 122.4, 125.3, 129.9, 134.1, 136.8, 148.5, 149.5, 154.6, 156.8, 161.3, 161.8. LRMS ASAP [**2b** + H^+^] 281.09; HRMS ASAP [**2b**]**^−^** calculated *m*/*z* C_16_H_12_N_2_O_3_ 280.0881, found = 280.0847.

#### 3.2.3. Synthesis of 2-oxo-*N*-(2-(pyridin-2-yl) ethyl)-2H-chromene-3-carboxamide (**2c**)

Yield of **2c**: 25%; white solid; FTIR (neat, cm^−1^): C=O 1706, N–H 3299 and 1566, C=N 1654, C–N 1516; ^1^H-NMR (500 MHz, DMSO-*d_6_*, ppm): *δ* 3.00 (2H, t, *J* = 7.0 Hz, CH_2_-7ʹ), 3.74 (2H, q, *J* = 6.6 Hz, CH_2_-8ʹ), 7.24 (1H, t, *J* = 4.8 Hz, CH-4ʹ), 7.31 (1H, d, *J* = 7.8 Hz, CH-3′), 7.43 (1H, t, *J* = 6.0 Hz, CH-7),7.51 (1H, d, *J* = 8.4 Hz, CH-8), 7.70–7.76 (2H, m, 5ʹ and 6), 7.98 (1H, d, *J* = 6.2 Hz, CH-5), 8.52 (1H, d, *J* = 3.8 Hz, CH-6ʹ), 8.87 (1H, s, CH-4), 8.92 (1H, t, *J* = 5.6 Hz, NH); ^13^C-NMR (100 MHz, DMSO-*d_6_*, ppm): *δ* 36.9, 38.8, 116.2, 118.5, 118.9, 121.7, 123.3, 125.2, 130.3, 134.1, 136.6, 147.6, 149.2, 153.9, 159.0, 160.4, 161.0. LRMS ASAP [**2c** + H^+^] 295.10; HRMS ASAP [**2c** + H^+^] calculated *m*/*z* C_17_H_15_N_2_O_3_ 295.1038, found = 295.1079.

#### 3.2.4. Synthesis of *N*-((1H-benzo[d]imidazol-2-yl) methyl)-2-oxo-2H-chromene-3-carboxamide (**2d**)

Yield of **2d**: 26%; orange solid; FT-IR (neat, cm^−1^): C=O 1691, N–H 3395 and 1566, C=N 1653, C–N 1544; ^1^H-NMR (400 MHz, DMSO-*d_6_*, ppm): *δ* 4.94 (2H, s, CH_2_), 6.63 (1H, t, *J* = 7.6 Hz, CH_benzoimidazol_) 6.80 (1H, d, *J* = 8.0 Hz, CH_benzoimidazol_), 6.96 (1H, t, *J* = 7.4 Hz, CH_benzoimidazol_), 7.46–7.57 (3H, m, CH-8, 7 and benzoimidazol), 7.78 (1H, t, *J* = 7.9 Hz, CH-6), 8.03 (1H, d, *J* = 7.7 Hz, CH-5), 8.95 (1H, s, CH-4), 10.10 (s, 1H, NH_amide_); ^13^C (100 MHz, DMSO-*d_6_*, ppm): *δ* 52.6, 116.3, 116.3, 116.5, 117.0, 118.6, 119.9, 123.4, 124.5, 125.4, 126.3, 130.5, 134.4, 141.5, 147.4, 154.7, 159.9, 163.3; LRMS ASAP [**2d** + H_2_O + H^+^] 338.34; HRMS ASAP [**2d**]^−^ calculated *m*/*z* C_18_H_13_N_3_O_3_ 319.0990, found = 319.0955.

#### 3.2.5. Synthesis of *N*-(2-aminoethyl)-2-oxo-2H-chromene-3-carboxamide (**2e**)

The crude product received by the reaction of coumarin-3-carboxylic acid and **3e** following the general procedure was purified by column chromatography on silica gel using 50% ethyl acetate in hexane to give desired product Boc-**2e** (yield 46%). Boc-**2e** (0.50 g, 1.5 mmol) was treated with trifluoroacetic acid (TFA, 5 mL) over an ice bath for 2 h. The volatiles were removed under vacuum, and the residues were taken up in water (10 mL). The mixture was neutralized with 1 M aqueous NaOH and extracted with DCM (50 mL), twice. The organic residues were washed with a saturated solution of Na_2_CO_3_ (aq) (50 mL), then brine (50 mL), and afterwards, dried over Na_2_SO_4_, filtered, and concentrated under reduced pressure to give **2e** (0.23 g, 1.0 mmol, 66%) as a white solid. Overall yield of **2e**: 30%; white solid; FT-IR (neat, cm^−1^): C=O 1678, N–H 3318 and 1566, C–N 1512; ^1^H-NMR (400 MHz, DMSO-*d_6_*, ppm): *δ* 2.98 (2H, t, *J* = 6.1 Hz, CH_2_-2ʹ), 3.55 (2H, t, *J* = 6.1 Hz, CH_2_-1ʹ) 7.46 (1H, t, *J* = 7.6 Hz, CH-6), 7.54 (1H, d, *J* = 8.4 Hz, CH-8), 7.74-7.89 (3H, m, CH-7 and NH_2_), 8.01 (1H, d, *J* = 7.8 Hz, CH-5), 8.89 (1H, s, CH-4), 8.93 (1H, t, *J* = 6.0 Hz, NH). ^13^C-NMR (100 MHz, DMSO-*d_6_*, ppm): *δ* 37.2, 38.5, 116.3, 118.5, 118.9, 125.4, 130.5, 134.4, 147.8, 154.0, 160.2, 162.1; LRMS ASAP [**2e** + H^+^] 233.09; HRMS ASAP [**2e**]^−^ calculated *m*/*z* C_12_H_12_N_2_O_3_ 232.0880, found = 232.0846.

#### 3.2.6. Synthesis of **2b**-Cu^2+^ Complex for IR Study

Coumarin **2b** was dissolved in acetonitrile, and then an aqueous solution of 0.5 or 1 equivalent of CuCl_2_ was added and left to stir until fully dissolved. The volatiles were reduced under vacuum to give the desired Cu^2+^ complexes used without further purification to record IR and mass spectra.

#### 3.2.7. The preparation of a Single Crystal of **2b** and **2b**–Cu^2+^ Complex

**2b** was dissolved in acetonitrile first and the crystal was grown under vapour diffusion into EtOH. One equivalent of **2b** and CuCl_2_ were dissolved in a solution of acetonitrile and water. The single crystal was grown under vapour diffusion into EtOH.

### 3.3. Optical Properties of **2a**–**e**

CuCl_2_ stock solution (1.00 mM) was prepared in aqueous solution of DMSO/HEPES buffer (20 mM, pH = 7) (*v*/*v*, 1/9), and stock solutions of **2a**–**e** (0.9 mM) were prepared in DMSO/HEPES buffer (20 mM, pH = 7) (*v*/*v*, 1/9).

#### 3.3.1. Fluorescence Intensity of **2a**–**e**

Fluorescence intensity of **2a**–**e** was recorded at pH 7 at concentrations in the range of 5–80 μM, diluted from the stock solution using DMSO/HEPES buffer on Varian Cary Eclipse spectrophotometer, with excitation and emission slit widths of 5 nm at their corresponding excitation maxima.

#### 3.3.2. pH Dependence of Fluorescence Intensity

Fluorescence intensities of **2a**–**e** (30 μM) with slit widths of 5 nm were recorded across the pH range of 3–13 at their corresponding excitation maxima.

#### 3.3.3. The Optical Properties of **2a**–**e** in the Presence of 1 eq. of Cu^2+^

UV-Vis and fluorescence spectra were recorded for **2a**–**e** (30 μM) and **2a**–**e** (30 μM) in the presence of CuCl_2_ (30 μM) in DMSO/HEPES buffer (20 mM, pH = 7) in the 250–600 nm region for the UV-Vis and with slit widths of 5 nm for fluorescence studies.

#### 3.3.4. Fluorescence Spectra of **2b** and **2d** in the Presence of Cu^2+^ at Various Concentrations

Fluorescence spectra of **2b** and **2d** (30 μM) were recorded in the presence of CuCl_2_ (0–80 μM) in DMSO/HEPES buffer (20 mM, pH = 7) with slit widths of 5 nm at their corresponding excitation maxima, respectively. The kinetics study was conducted using 100 μL of 60 μM solutions of compound **2b** and **2d** in DMSO/HEPES buffer (20 mM, pH = 7, *v*/*v*, 1/9) with the automatic pipetting of 100 μL of 120 μM Cu^2+^ solution to form the complexes of **2b**–Cu^2+^ and **2d**–Cu^2+^ at 25 °C. After 3.5 s shaking time, the changes in time of the fluorescence for **2b**–Cu^2+^ and **2d**–Cu^2+^ complexes were recorded on CLARIOstar plate reader.

#### 3.3.5. Interference Studies

The fluorescence intensities of **2b** and **2d** (30 μM) were recorded in the presence of following metal chlorides: Al^3+^, Ca^2+^, Cd^2+^, Co^2+^, Fe^3+^, Mn^2+^, Pb^2+^, Hg^2+^, Ni^2+^, Fe^2+^, Cu^+^, Mg^2+^, and Zn^2+^ (20 eq., 600 μM) in DMSO/HEPES buffer (20 mM, pH = 7), both in the absence and presence of CuCl_2_ (30 μM). Firstly, an aliquot of selected potentially interfering metal ions (60 μM, 1 mL) and **2b** or **2d** (60 μM, 1 mL) was mixed together to reach a final concentration of 30 μM. The fluorescence of this solution was recorded. Then, a stock solution of CuCl_2_ (60 μL) was added to those, and optical properties were again measured. Fluorescence spectra were recorded with slit widths of 5 nm at their corresponding excitation maxima.

#### 3.3.6. Fluorescence Signal Recovery Study

The fluorescence intensity of **2b** (30 μM) was recorded in DMSO/HEPES buffer (20 mM, pH = 7) upon the alternating addition of 1 equivalent of CuCl_2_, followed by 1 equivalent of ethylene diamine tetraacetic acid (EDTA) in repeat cycles. 

#### 3.3.7. Cu^2+^ Sensing in Aqueous Soil Extracts

Soil samples were collected from the roadside in the western parts of Melbourne, Victoria, Australia. Soil samples (2.5 g) were dispersed in water (25 mL) and shaken for one week to extract water-soluble copper. The extracts were filtered through a 0.45 mm pore-size nylon filter membrane. Each extract was analysed for their copper content via ICP-MS [37] and by recording the fluorescence quenching using 60 µM solution of **2b** (1 mL in DMSO/HEPES, *v*/*v*, 2:8) mixed with the soil extract (and 1 mL of water) for the quantitation of Cu^2+^ in soil using a pre-recorded standard curve.

## 4. Conclusions

A series of five coumarin derivatives were systematically designed, synthesized, and evaluated for their potential use for Cu^2+^-sensing in aqueous media. Coumarins **2b** and **2d** exhibited selectivity towards Cu^2+^ in the presence of other multivalent metal ions. The linear ranges of the sensing signal versus Cu^2+^ concentration were 0–80 μM for **2b**, and 0–10 μM for **2d**, falling into the relevant and desired concentration range for environmental sensing techniques. Limits of detection in aqueous media were 0.14 μM and 0.38 μM for **2b** and **2d**, respectively, satisfactory for the potential application of these molecular optical probes. Probe **2b** exhibited superior applicable sensing performance towards Cu^2+^ when compared to the other coumarins presented in this work, hence it was further studied to gain understanding on its interaction with Cu^2+^. Solid state ATR-FTIR, single crystal X-ray diffraction, and mass spectrometry, revealed a 1:1 stoichiometric complex as the responsible species for the displayed quenching of the fluorescence of **2b**. The formation of this coordination complex resulted in a Cu^2+^-concentration dependent sensing signal, while offered good recovery of the probe signal based on the fluorescence recovery study using EDTA. As a proof-of-concept for its applicability, **2b** provided results in the Cu^2+^ quantitation of soil extracts which were comparable with the standard ICP-MS method. Furthermore, by assessing structurally related coumarins **2a**–**e** (owning slightly different Cu^2+^-coordinating moieties), we gained valuable information on the required size of the coordination-site, relative spatial and atomic arrangement, and electron distribution of the coordinating heteroatoms. The fluorescence responses of **2a**–**c** suggested that the size of the coordination site has a significant impact on the Cu^2+^ coordination. Finally, the fluorescence titration of **2b** and **2d** suggested that the lone pair’s availability of the heteroatoms at the same relative position also affects the sensing properties, both in coordination speed and selectivity. These results will be used in our future work for the systematic design and development of metal-ion coordinating optical sensing probes, owning enhanced selectivity and sensitivity. 

## Figures and Tables

**Table 1:** Selected amines **3a**–**e**, synthesized coumarin derivatives **2a**–**e**, and their corresponding yields (%), absorption maxima, excitation and emission maxima, and Stokes shifts.

	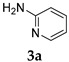	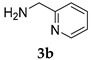	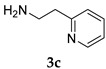	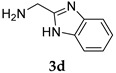	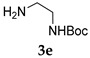
Probe	2a	2b	2c	2d	2e^*^
Yield %	26	23	25	26	30^^^
λ_abs_ (nm)	298	300	300	306	300
λ_ex_ (nm)	280	303	303	325	303
λ_em_ (nm)	406	412	412	436	412
Δν (nm)	126	109	109	111	109

^*^2e was obtained after removal of the protecting group of Boc-**2e** using TFA in DCM to give **2e** (see Section 3.2.5); ^^^ overall yield.

**Table 2:** A comparison of sensing properties among **2b**, **2d**, and **1a.**

	2b	2d	1a*
Limit of detection (μM)	0.14	0.38	0.5
Linear range (μM)	0–80	0–10	0–50
Interference	Fe^3+^, Cd^2+^	Co^2+^, Fe^3+^, Ni^+^, Hg^2+^	No data included
Association constant (M^−1^)	6.85 × 10^4^	7.83 × 10^5^	1.17 × 10^5^

* Values taken from literature [20].

**Table 3:** Characteristic ATR-FTIR peaks and their intensities (in brackets) for **2b** before and after coordination of Cu^2+^.

	ν(C=O) Coumarin	ν(C=O) Amide	ν(C=N) Pyridine	ν(C=N) Pyridine	ν(N-H) Amide	ν(C-N) Amide	ν(C=C) Pyridine	ν(C=C) Pyridine
**2b**	1706 (0.74)	1648 (0.47)	1608 (0.47)	1601 (0.47)	1564 (0.62)	1514 (0.85)	1480 (0.44)	1451 (0.52)
+0.5 eq. Cu^2+^	1707 (0.30)	1653 (0.75)	1609 (0.59)	1595 (0.54)	1566 (0.49)	1535 (0.36)	1483 (0.34)	1451 (0.40)
+1eq. Cu^2+^	1707 (0.15)	1653 (0.71)	1610 (0.69)	1595 (0.51)	1567 (0.46)	1535 (0.57)	1483 (0.36)	1451 (0.39)

**Table 4:** Experimental and literature references of bond lengths (Å).

	In **2b**	In **2b**-Cu^2+^	Single bond length
Coumarin C=O	1.21	1.35	1.43

**Table 5:** Determination and recovery values of Cu^2+^ in soil samples using both ICP-MS (μM) and **2b** (μM).

Sample	Cu^2+^ Concentration by ICP-MS (μM)	Fluorescence of Solution of 2b at λ_ex_ = 412 nm (a.u.)	Cu^2+^ Concentration by Using 2b (μM)	Recovery
1	1.23 ± 0.13	173.54 ± 0.69	1.03 ± 0.13	83.7%
2	1.36 ± 0.21	175.11 ± 0.36	1.27 ± 0.05	93.4%
3	1.71 ± 0.27	178.48 ± 0.69	1.75 ± 0.09	102.3%

## Supplementary Materials

The following are available online at https://www.mdpi.com/1420-3049/24/19/3569/s1, Figure S1: Figure S1 ^1^H NMR spectrum of **2a**, Figure S2 ^13^C NMR spectrum of **2a**, Figure S3 IR spectrum of **2a**, Figure S4 High resolution mass spectra of **2a**, Figure S5 ^1^H NMR spectrum of **2b**, Figure S6 ^13^C NMR spectrum of **2b**, Figure S7 IR spectrum of **2b**, Figure S8 High resolution mass spectra of **2b**, Figure S9 ^1^H NMR spectrum of **2c**, Figure S10 ^13^C NMR spectrum of **2c**, Figure S11 spectrum of **2c**, Figure S12 High resolution mass spectra of **2c**, Figure S13 ^1^H NMR spectrum of **2d**, Figure S14 ^13^C NMR spectrum of **2d**, Figure S15 IR spectrum of **2d**, Figure S16 High resolution mass spectra of **2d**, Figure S17 ^1^H NMR spectrum of **2e**, Figure S18 ^13^C NMR spectrum of **2e**, Figure S19 IR spectrum of **2e**, Figure S20 High resolution mass spectra of **2e**, Figure S21 Uv-Vis absorbance **2a-e** (30 µM) in DMSO/HEPES buffer (*v*/*v*, 1/9) in the absence (black lines) and presence (red lines) of 1 equivalent of CuCl_2_: (a) **2a**; (b) **2b**; (c) **2c**; (d) **2d**; (e) **2e**, Figure S22 Kinetics study of (a) **2b** (λ_ex_=303 nm) and (b) **2d** (λ_ex_=325 nm) (30 µM) with the addition of 2 eq. of CuCl_2_ in HEPES/DMSO buffer, Figure S23 Fluorescence emission intensities of (a) **2b** (λ_ex_=303 nm) and (b) **2d** (λ_ex_=325 nm) both 30 μM in the presence (red lines) and absence (black lines) of Cu^2+^ with 20 equivalents of various cations in DMSO/HEPES buffer, Table S1 Fluorescence intensity variations of **2b** with adding 20 eq. metal ions, Table S2 Fluorescence intensity variations of **2d** with adding 20 eq. metal ions, Figure S24 LRMS of **2b**-Cu^2+^, Figure S25 Normalized IR spectra of **2b** with different ratios of Cu^2+^, Figure S26 Single crystal structure of **2b**, Figure S27 Standard curve for Cu^2+^ sensing by **2b** in DMSO/HEPES buffer (v/v, 1/9, 20 mM, pH=7), Figure S28 Fluorescence background for soil extracts in DMSO/HEPES buffer (v/v, 1/9, 20 mM, pH=7), Table S3 Crystal data and structure refinement for **2b**, Table S4 Crystal data and structure refinement for **2b**-Cu^2+^.
