# Supplementary material for: The Design and Synthesis of Fluorescent Coumarin Derivatives and Their Study for Cu2+ Sensing with an Application for Aqueous Soil Extracts
Source: Molecules. 2019 Oct 2;24(19):3569. doi: 10.3390/molecules24193569 (PMC6804054; doi:10.3390/molecules24193569)
Supplement: Supplementary file 1 [file molecules-24-03569-s001.pdf]

Supporting information

Design and synthesis of fluorescent coumarin derivatives and their study for Cu^{2+} sensing with an application on aqueous soil extracts

Bin Qian [1], Linda Váradi[1,2*], Adrian Trinchi[2], Suzie Reichman[1], Lei Bao[1], Minbo Lan[3], Gang Wei[4], Ivan Cole[1]

[1] School of Engineering, RMIT University, GPO Box 2476, Melbourne, Victoria, 3001, Australia;
[2] CSIRO Manufacturing, Bayview Avenue, Clayton, VIC, 3169, Australia;
[3] Shanghai Key Laboratory of Functional Materials Chemistry, School of Chemistry and Molecular Engineering, East China University of Science and Technology, 130 Meilong Road, Shanghai, 200237, China;
[4] CSIRO Mineral Resources, PO Box 218, Lindfield, NSW 2070, Australia;

* Correspondence: linda.varadi@rmit.edu.au; +61-03- 99253553

Table of content

1. Characterization of 2a 2

2. Characterization of 2b 3

3. Characterization of 2c 5

4. Characterization of 2d 7

5. Characterization of 2e 9

6. Uv-Vis absorbance for 2a-e with and without 1 eq. Cu^{2+} 12

7. Kinetics study of 2b and 2d 12

8. Interference study of 2b and 2d 13

9. Mass spectra of 2b-Cu^{2+} 14

10. IR spectra of 2b with Cu^{2+} 15

11. Soil tests 16

12. Single crystal data for 2b and 2b-Cu^{2+} 16

1. Characterization of 2a

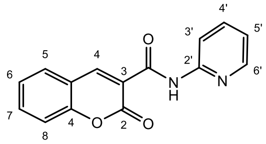

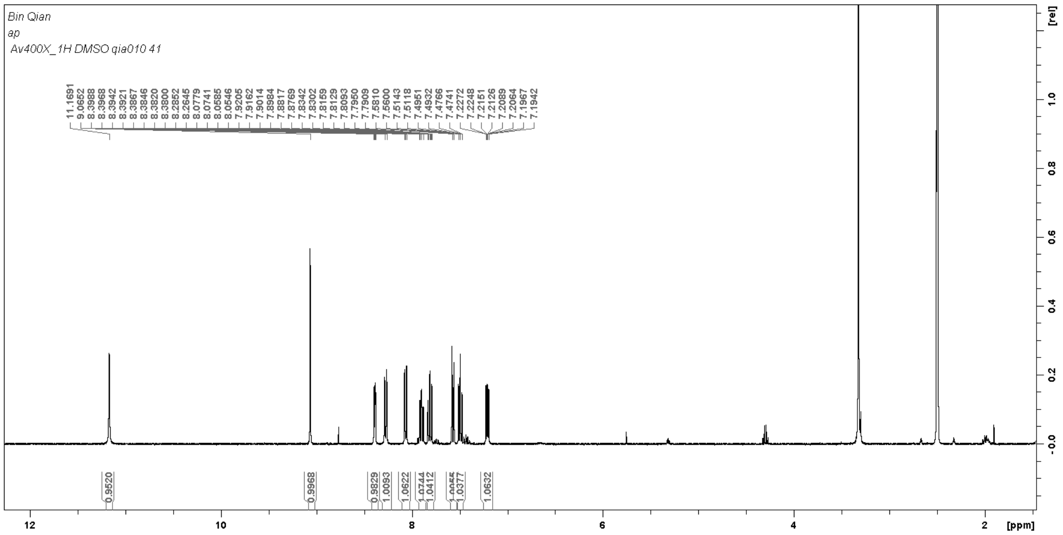

Figure S1 ^{1}H NMR spectrum of **2a**

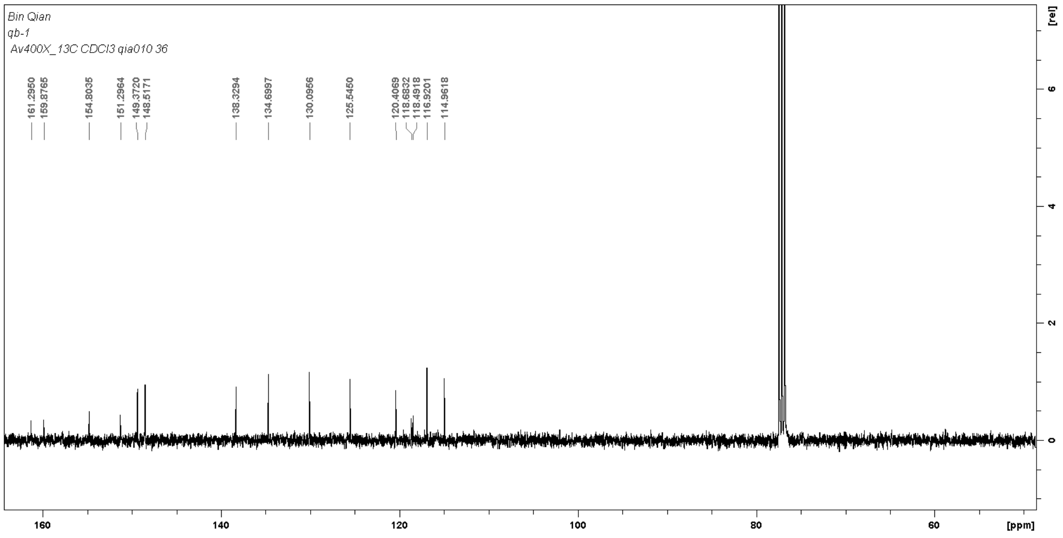

Figure S2 ^{13}C NMR spectrum of **2a**

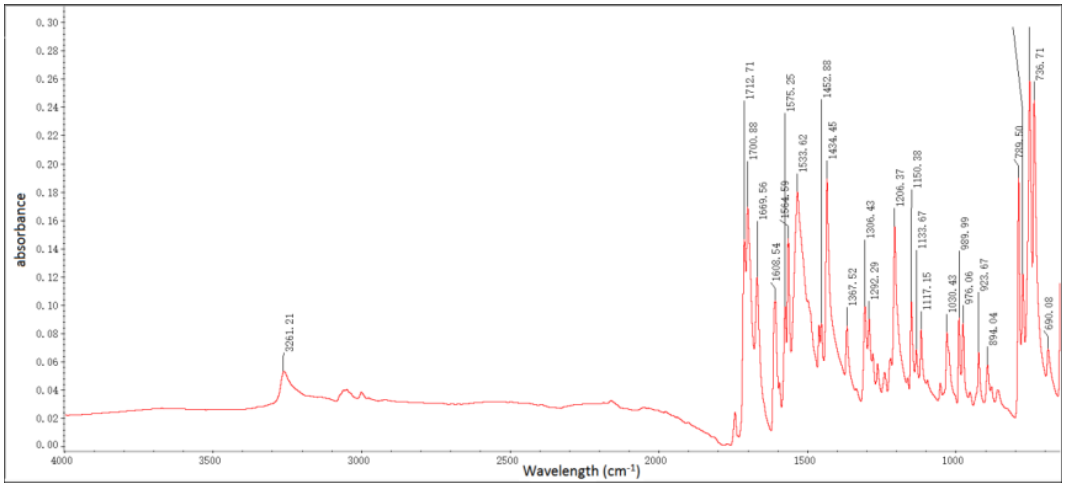

Figure S3 IR spectrum of **2a**

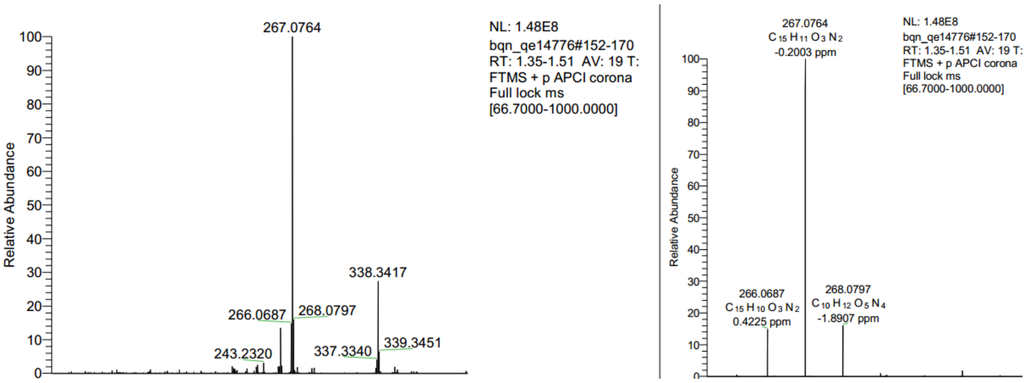

Figure S4 High resolution mass spectra of **2a**

2. Characterization of 2b

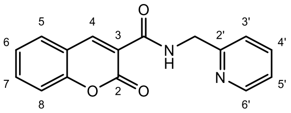

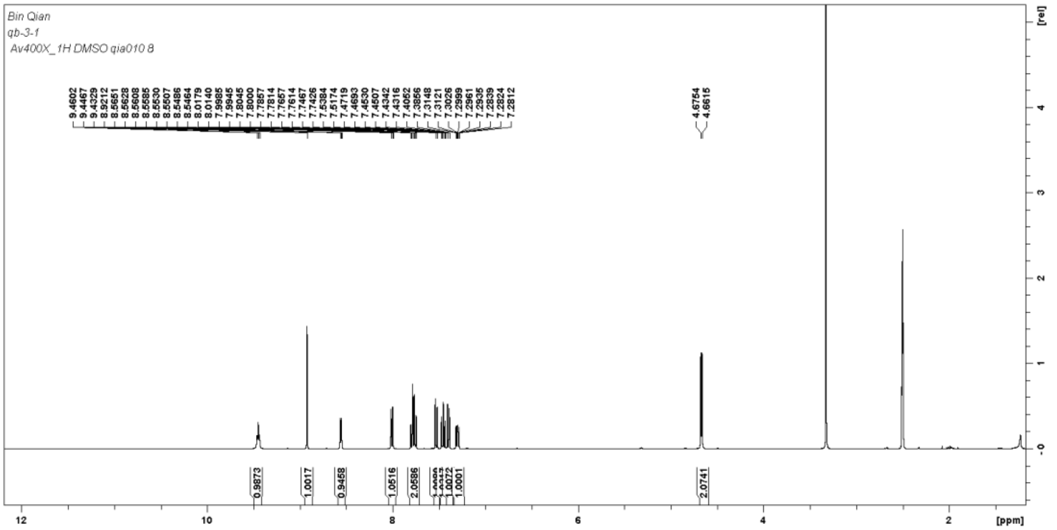

Figure S5 ¹H NMR spectrum of **2b**

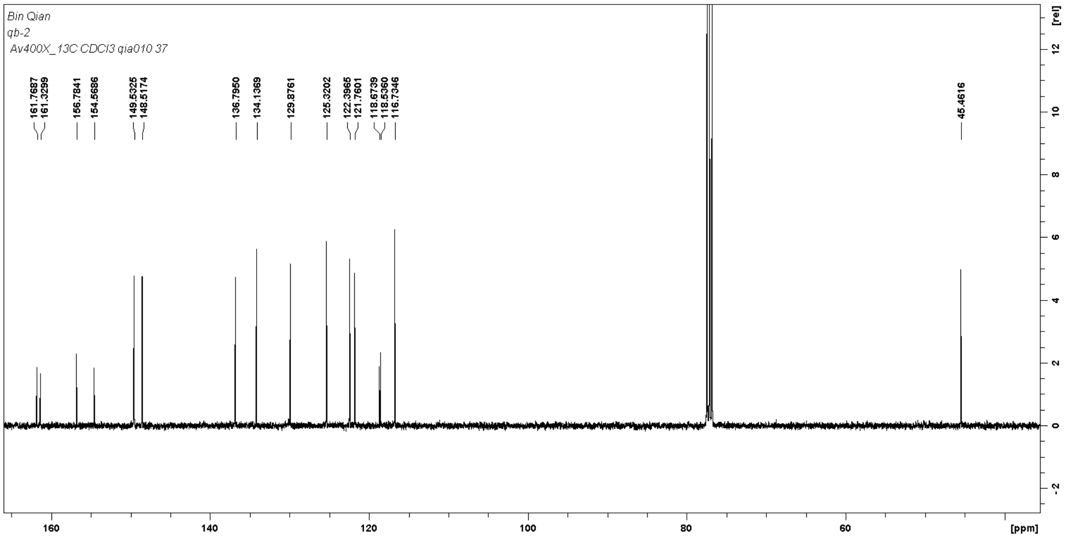

Figure S6 ¹³C NMR spectrum of **2b**

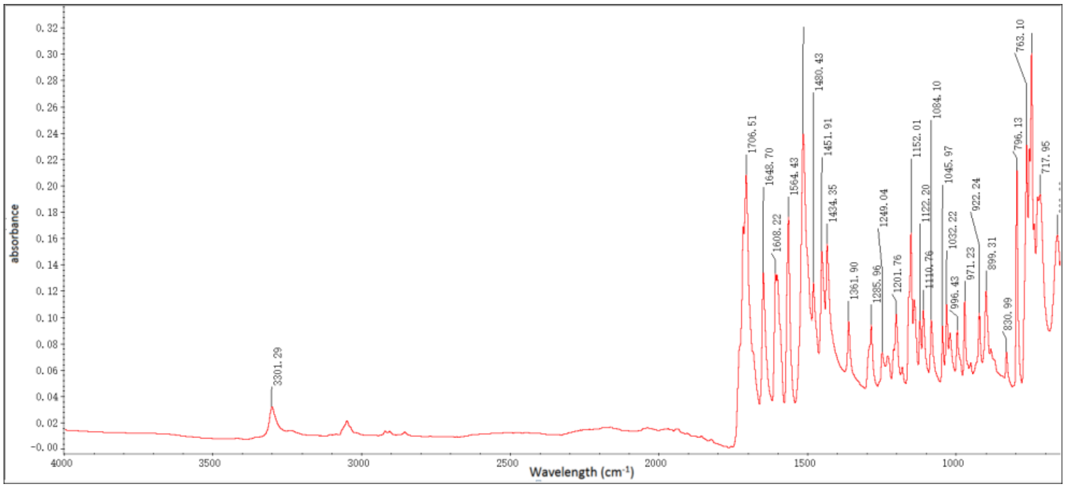

Figure S7 IR spectrum of **2b**

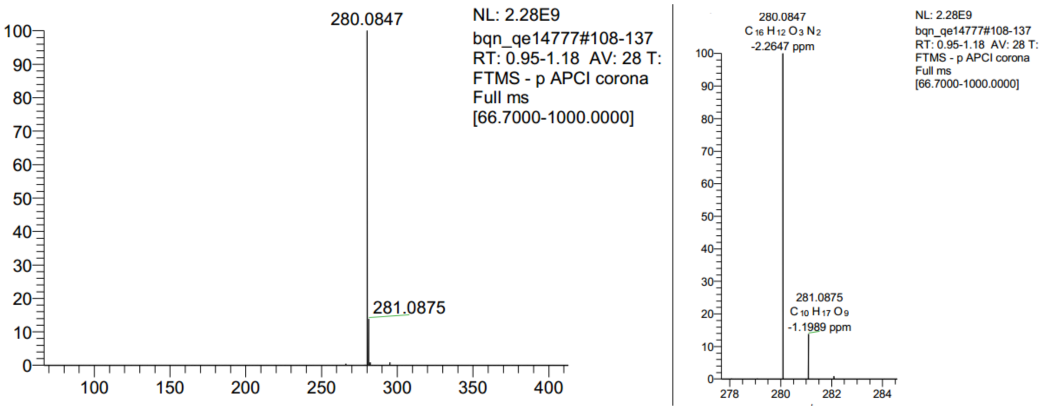

Figure S8 High resolution mass spectra of **2b**

3. Characterization of 2c

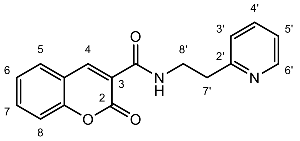

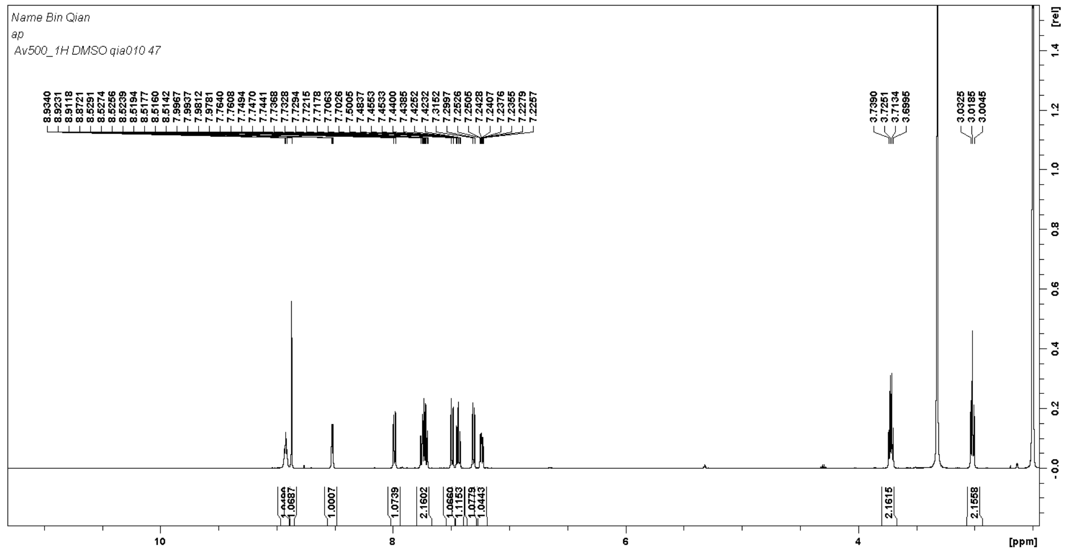

Figure S9 ^{1}H NMR spectrum of **2c**

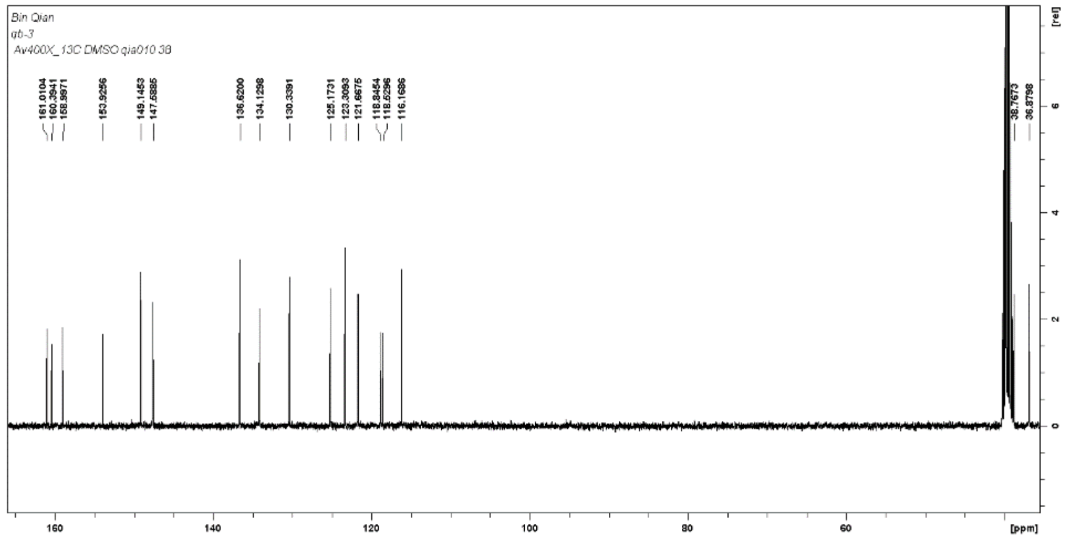

Figure S10 ^{13}C NMR spectrum of **2c**

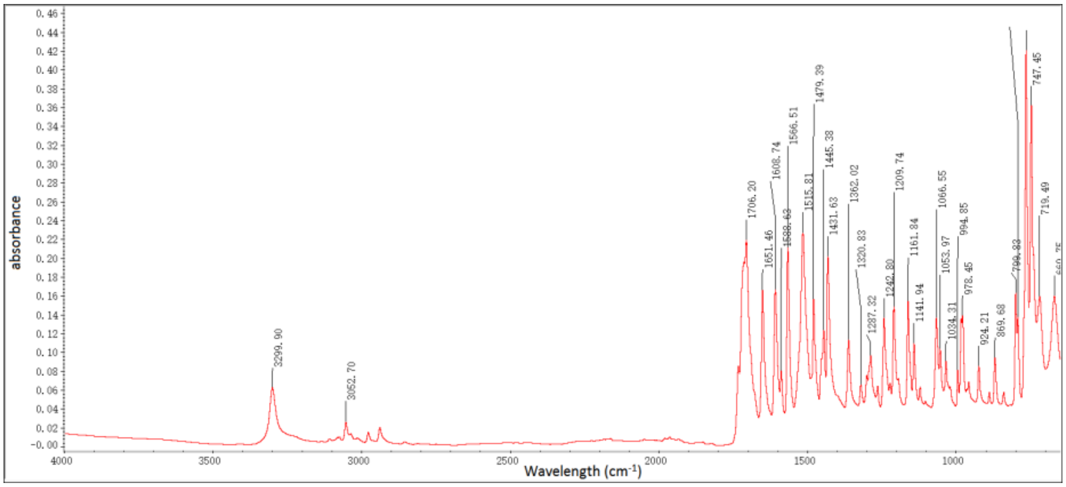

Figure S11 spectrum of **2c**

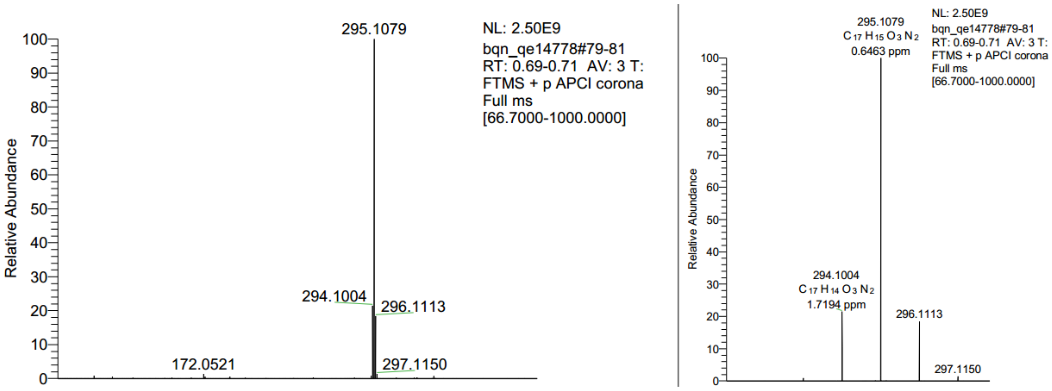

Figure S12 High resolution mass spectra of **2c**

4. Characterization of 2d

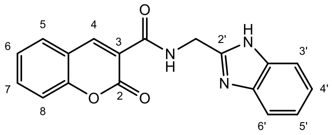

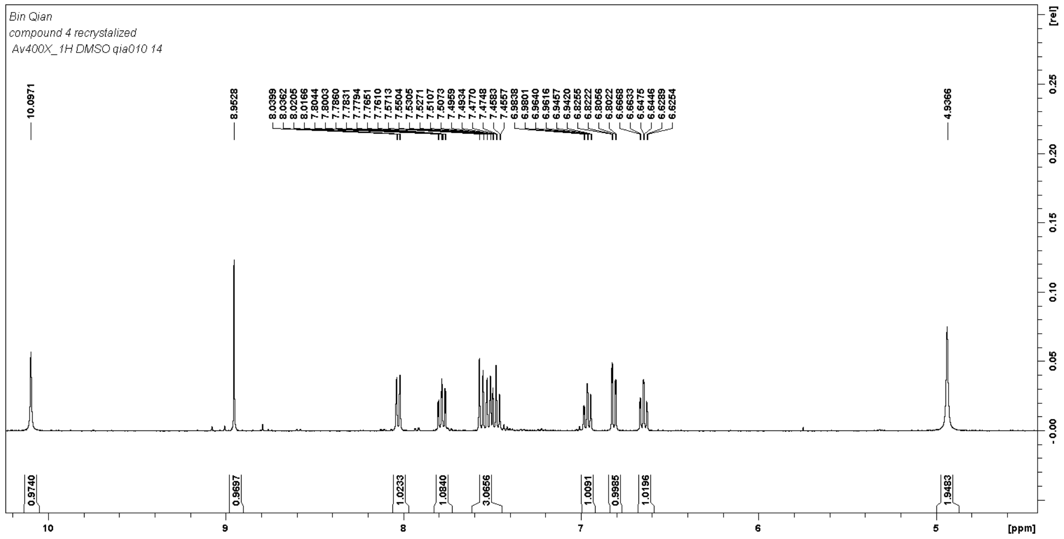

Figure S13 ^{1}H NMR spectrum of **2d**

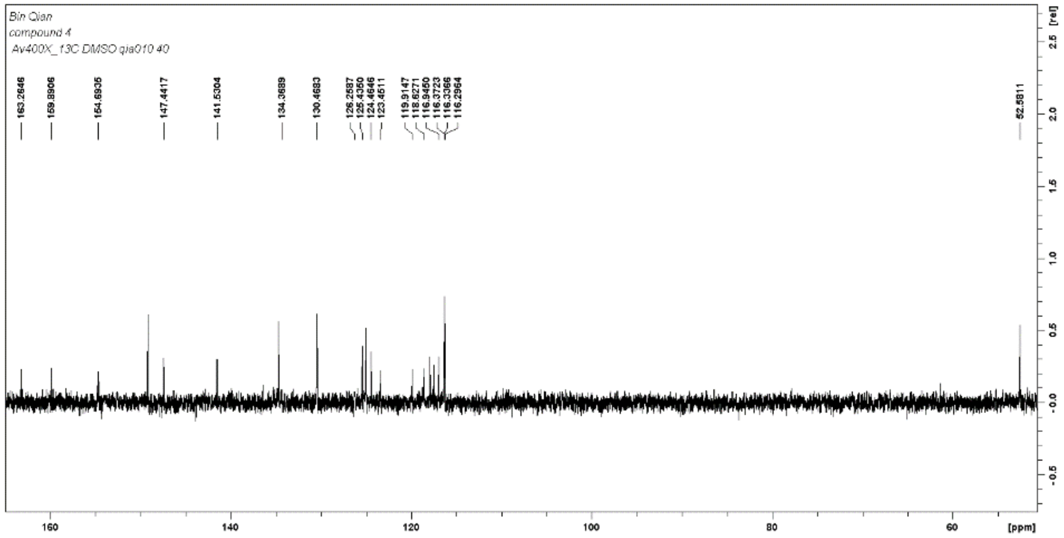

Figure S14 ^{13}C NMR spectrum of **2d**

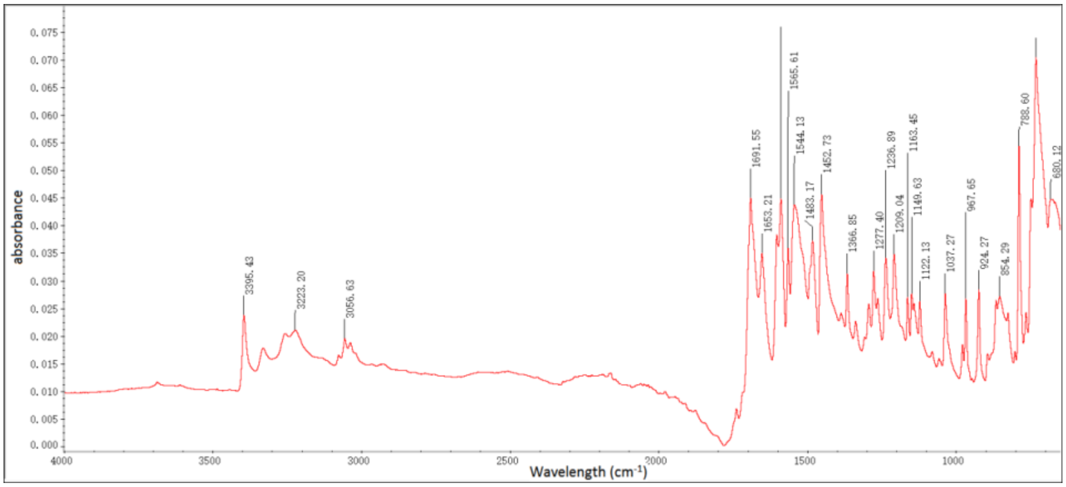

Figure S15 IR spectrum of **2d**

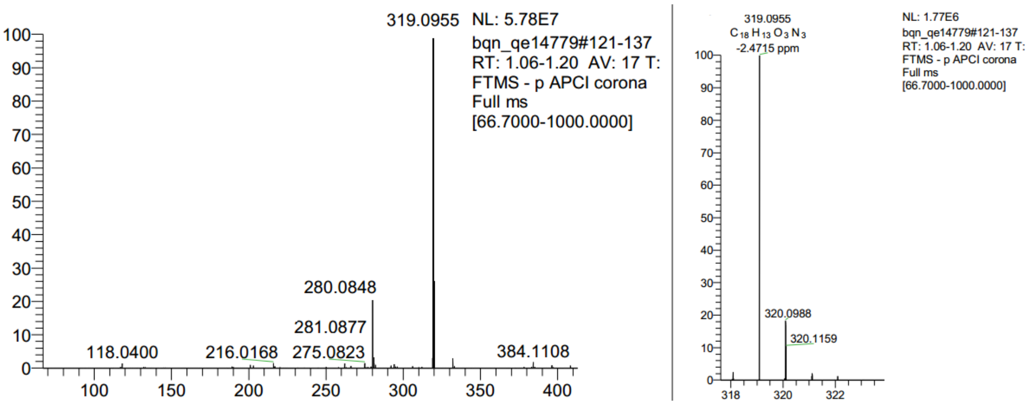

Figure S16 High resolution mass spectra of **2d**

5. Characterization of 2e

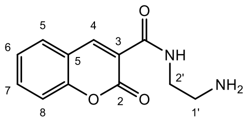

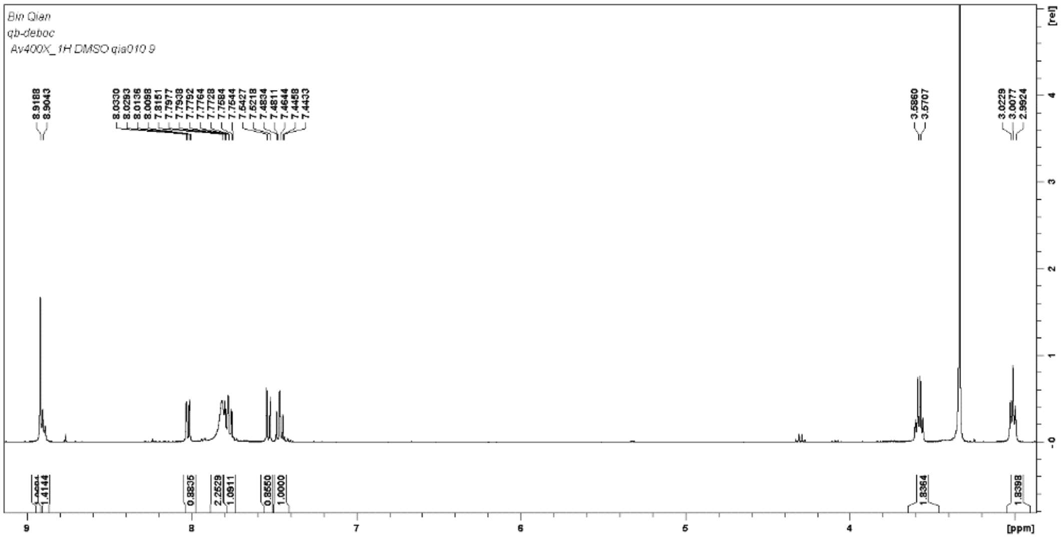

Figure S17 ^{1}H NMR spectrum of **2e**

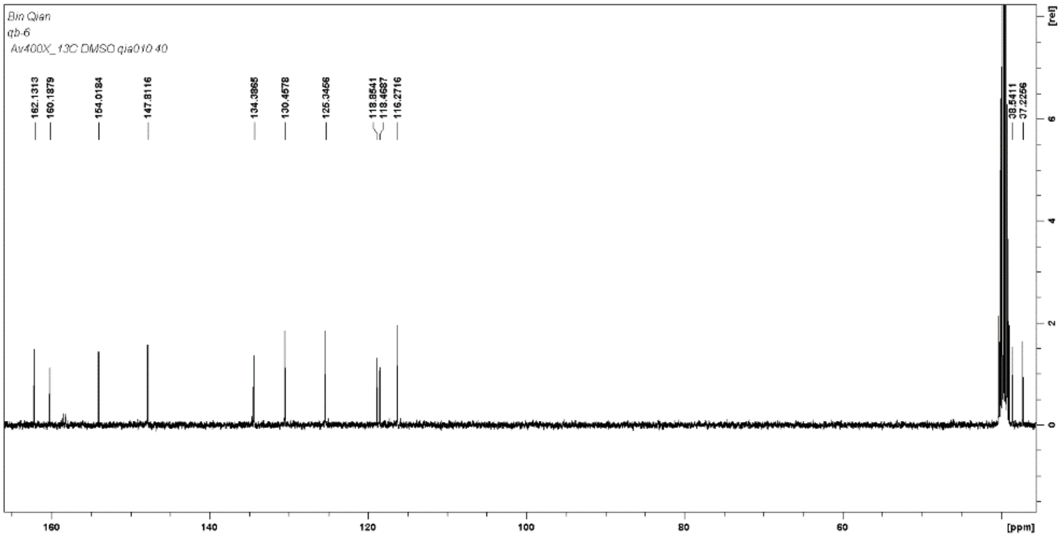

Figure S18 ^{13}C NMR spectrum of **2e**

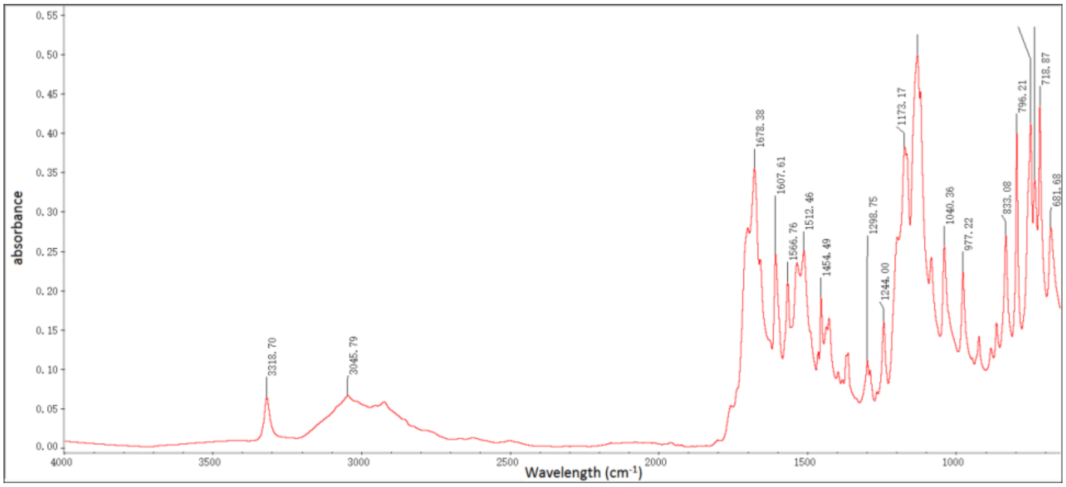

Figure S19 IR spectrum of **2e**

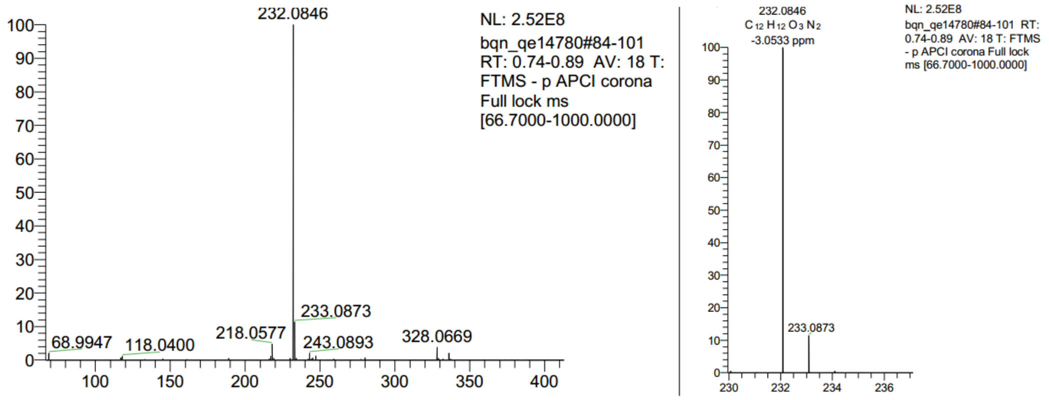

Figure S20 High resolution mass spectra of **2e**

6. UV-Vis absorbance for 2a-e with and without 1 eq. Cu^{2+}

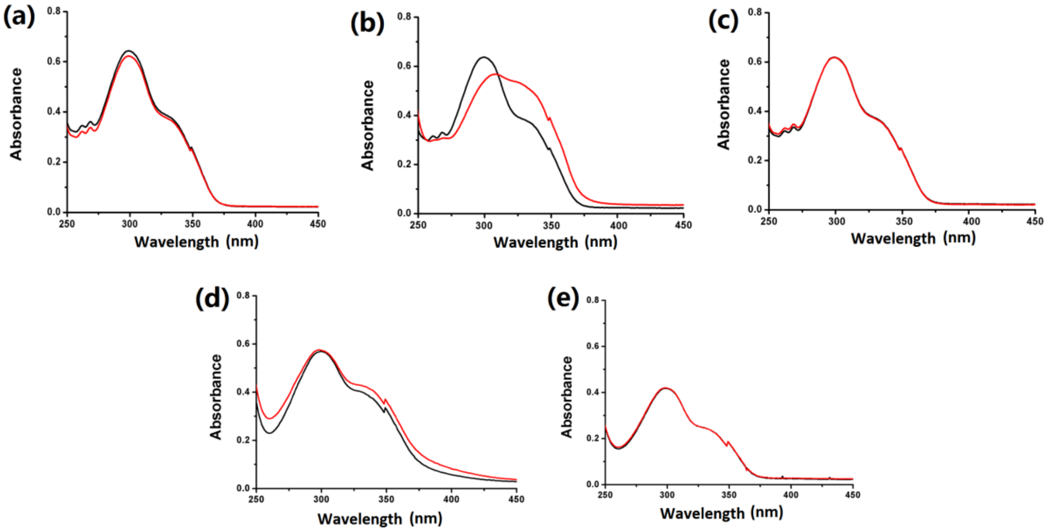

Figure S21 Uv-Vis absorbance **2a-e** (30 μM) in DMSO/HEPES buffer (v/v, 1/9) in the absence (black lines) and presence (red lines) of 1 equivalent of $CuCl_2$: (a) **2a**; (b) **2b**; (c) **2c**; (d) **2d**; (e) **2e**

7. Kinetics study of 2b and 2d

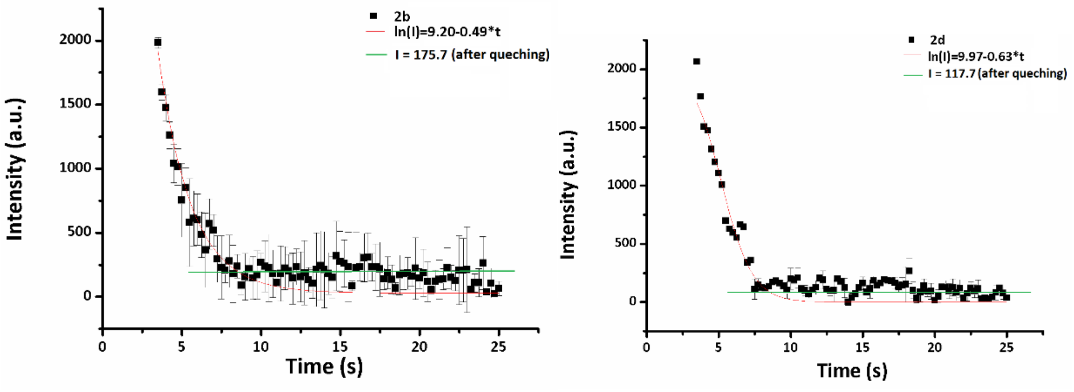

(a) (b)

Figure S22 Kinetics study of (a) **2b** (λ_{ex}=303 nm) and (b) **2d** (λ_{ex}=325 nm) (30 μM) with the addition of 2 eq. of $CuCl_2$ in HEPES/DMSO buffer

8. Interference study of 2b and 2d

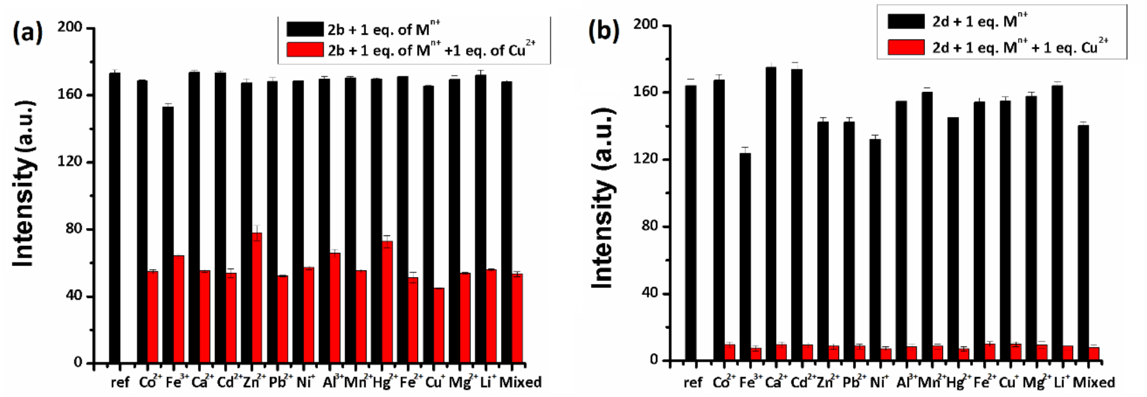

Figure S23 Fluorescence emission intensities of (a) **2b** (λ_{ex}=303 nm) and (b) **2d** (λ_{ex}=325 nm) both 30 μM in the presence (red lines) and absence (black lines) of Cu^{2+} with 20 equivalents of various cations in DMSO/HEPES buffer.

Table S1 Fluorescence intensity variations of **2b** with adding 20 eq. metal ions

2b	Intensity (a.u.) after adding 20 eq. metal ions	ΔIntensity	Δ%
ref	168.6245		
Cu^{2+}	50.408		
Co^{2+}	160.9087	7.71575	4.58%
Fe^{3+}	144.838	23.78648	14.11%
Ca^{2+}	159.633	8.99144	5.33%
Cd^{2+}	151.3966	17.22786	10.22%
Zn^{2+}	160.2103	8.41412	4.99%
Pb^{2+}	164.7546	3.8699	2.29%
Ni^{+}	155.4868	13.13762	7.79%
Al^{3+}	154.2941	14.33038	8.50%
Mn^{2+}	157.4268	11.19762	6.64%
Hg^{2+}	159.9028	8.72169	5.17%
Fe^{2+}	165.4378	3.18669	1.89%
Cu^{+}	154.2277	14.39674	8.54%
Mg^{2+}	166.3161	2.30838	1.37%
Li^{+}	162.8126	5.81187	3.45%
Mixed	151.2277	17.39674	10.32%

Table S2 Fluorescence intensity variations of **2d** with adding 20 eq. metal ions

2d	Intensity (a.u.) after adding 20 eq. metal ions	ΔIntensity	Δ%
ref	164.274		
Cu^{2+}	7.43938		
Co^{2+}	124.0102	40.2638	24.51%
Fe^{3+}	80.86041	83.41356	50.78%

Ca^{2+}	150.4194	13.85461	8.43%
Cd^{2+}	159.4749	4.79904	2.92%
Zn^{2+}	159.7672	4.50674	2.74%
Pb^{2+}	151.5827	12.6913	7.73%
Ni^{+}	131.5143	32.75965	19.94%
Al^{3+}	154.5642	9.70979	5.91%
Mn^{2+}	160.1416	4.1324	2.52%
Hg^{2+}	128.713	35.561	21.65%
Fe^{2+}	156.2374	8.03659	4.89%
Cu^{+}	149.3737	14.90027	9.07%
Mg^{2+}	155.7565	8.51744	5.18%
Li^{+}	155.7248	8.54913	5.20%
Mixed	134.0102	30.2638	18.42%

9. Mass spectra of 2b-Cu^{2+}

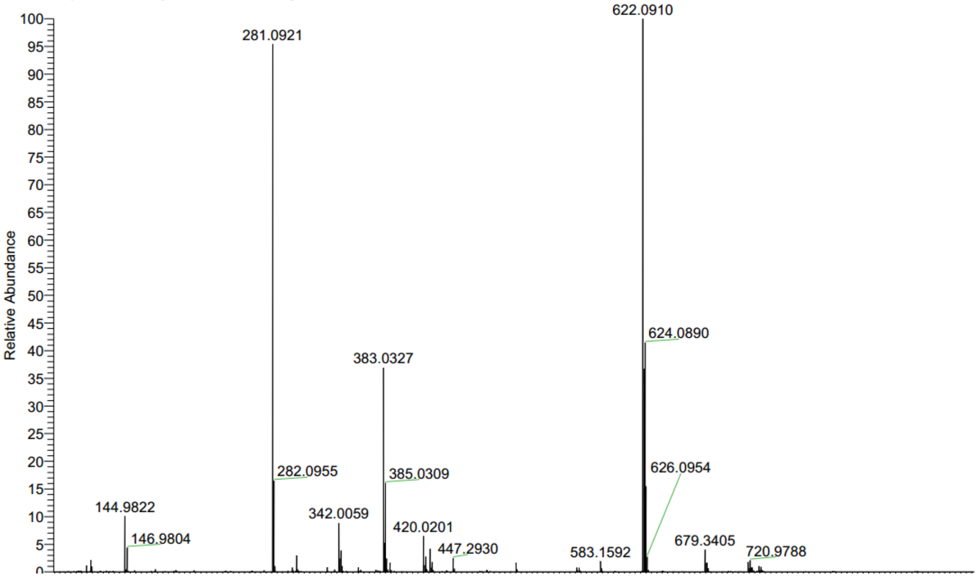

Figure S24 LRMS of **2b**-Cu^{2+}

10. IR spectra of 2b with Cu^{2+}

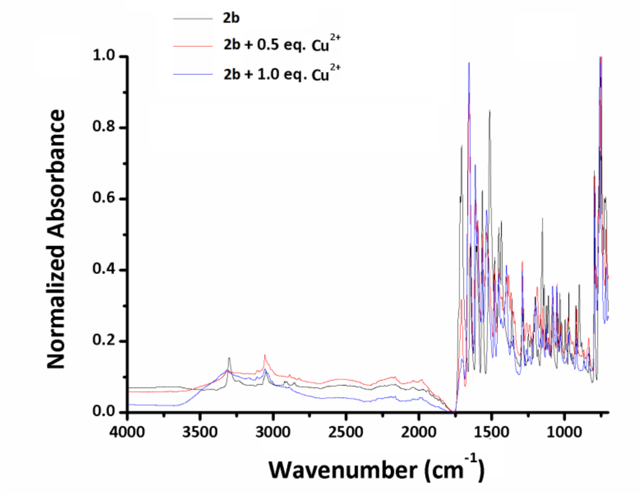

Figure S25 Normalized IR spectra of **2b** with different ratios of Cu^{2+}

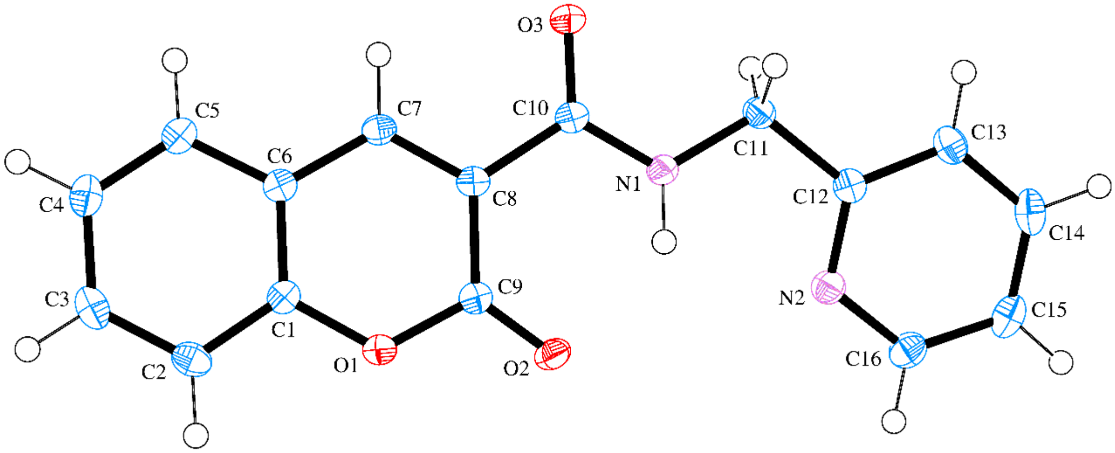

Figure S26 Single crystal structure of **2b**

11. Soil tests

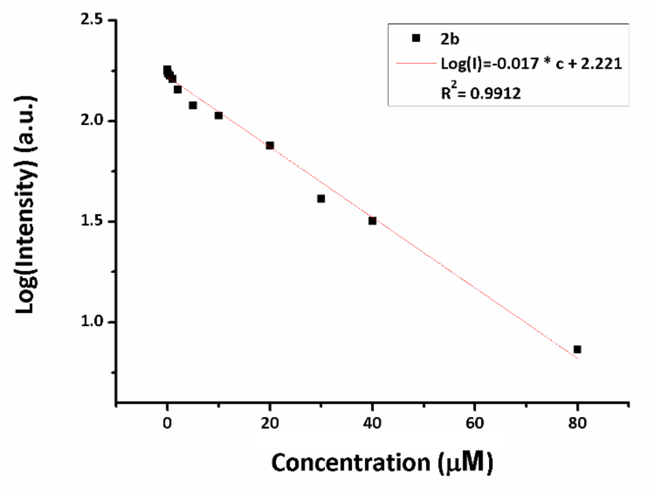

Figure S27 Standard curve for Cu^{2+} sensing by **2b** in DMSO/HEPES buffer (v/v, 1/9, 20 mM, pH=7)

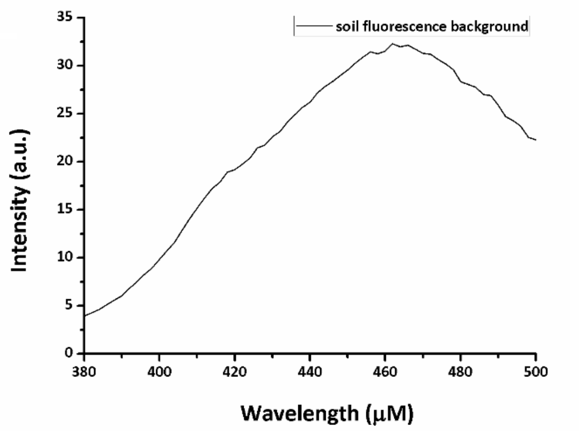

Figure S28 Fluorescence background for soil extracts in DMSO/HEPES buffer (v/v, 1/9, 20 mM, pH=7)

12. Single crystal data for 2b and **2b**-Cu^{2+}

Table S3 Crystal data and structure refinement for **2b**

**Formula**		$C_{16}H_{12}N_2O_3$		
**Formula weight**		280.28		
**Crystal system**		monoclinic		
**Crystal size (mm³)**		0.388 x 0.046 x 0.034		
**Space group**		P 21/c		
**Unit cell dimensions**	**a (Å)**	5.0235 (5)	**α°**	90

	**B (Å)**	21.729 (2)	**β°**	7.960 (2)
	**c (Å)**	12.0529 (12)	**γ°**	90
**Volume (Å^3)**		1303.0(2)		
**Z**		4		
**Theta range for data collection (°)**		1.874 to 27.499		
**Index ranges**		-6<=h<=6, -23<=k<=28, -15<=l<=15		
**Reflections collected**		12755		
**Refinement method**		Full-matrix least-squares on F^2		
**Data / restraints / parameters**		2994 / 0 / 193		
**Goodness-of-fit on F2**		1.037		
**Calculated density (Mg/cm^3)**		1.429		
**Absorption coefficient (mm^{-1})**		0.101		
**F(000)**		584		
**Max. and min. transmission**		0.746 and 0.714		
**Goodness-of-fit on F2**		0.565		
**Final R indices**	**I>2σ(I)**	R1 = 0.0365, wR2 = 0.0984		
	**all data**	R1 = 0.0433, wR2 = 0.1027		

Table S4 Crystal data and structure refinement for **2b**-Cu^{2+}

**Formula**		$C_{16}H_{11}ClCuN_2O_3$		
**Formula weight**		378.26		
**Crystal system**		triclinic		
**Crystal size (mm)**		0.13 x 0.20 x 0.20		
**Space group**		P -1		
**Unit cell dimensions**	**a (Å)**	8.2691(14)	**α°**	92.736(4)
	**B (Å)**	8.9756(15)	**β°**	91.476(4)
	**c (Å)**	9.4832(16)	**γ°**	99.030(3)
**Volume (Å^3)**		693.9(2)		
**Z**		2		
**Theta range for data collection (°)**		2.15 to 26.44		
**Index ranges**		-10<=h<=10, -11<=k<=11, -11<=l<=11		
**Reflections collected**		9247		
**Refinement method**		Full-matrix least-squares on F^2		
**Data / restraints / parameters**		2839 / 0 / 208		
**Goodness-of-fit on F2**		0.565		
**Calculated density (Mg/cm^3)**		1.810		
**Absorption coefficient (mm^{-1})**		1.783		

**F(000)**		382
**Reflections collected**		9247
**Max. and min. transmission**		0.8053 and 0.7169
**Data / restraints / parameters**		2839 / 0 / 208
**Goodness-of-fit on F2**		0.565
**Final R indices**	**I>2σ(I)**	R1 = 0.0214, wR2 = 0.1102
	**all data**	R1 = 0.0218, wR2 = 0.1127
